# Impact of frailty on clinical outcomes in patients with and without COVID-19 pneumonitis admitted to intensive care units in Australia and New Zealand: a retrospective registry data analysis

**DOI:** 10.1186/s13054-022-04177-9

**Published:** 2022-10-03

**Authors:** Ashwin Subramaniam, Kiran Shekar, Christopher Anstey, Ravindranath Tiruvoipati, David Pilcher

**Affiliations:** 1 Department of Intensive Care, Frankston Hospital, Peninsula Health, Frankston, VIC 3199 Australia; 2 Peninsula Clinical School, Monash University, Frankston, VIC Australia; 3 Australian and New Zealand Intensive Care Research Centre (ANZIC-RC), School of Public Health and Preventive Medicine, Monash University, Melbourne, VIC Australia; 4 Adult Intensive Care Services, The Prince Charles Hospital, Brisbane, QLD Australia; 5 University of Queensland, Brisbane, QLD Australia; 6 Queensland University of Technology Brisbane and Bond University, Gold Coast, QLD Australia; 7 Griffith University, Gold Coast, QLD Australia; 8 Department of Intensive Care, Alfred Hospital, Melbourne, VIC Australia; 9 Centre for Outcome and Resource Evaluation, Australian and New Zealand Intensive Care Society, Melbourne, VIC Australia

**Keywords:** Frailty, Clinical Frailty Scale, CFS, COVID-19, Pandemic, ANZICS-APD

## Abstract

**Background:**

It is unclear if the impact of frailty on mortality differs between patients with viral pneumonitis due to COVID-19 or other causes. We aimed to determine if a difference exists between patients with and without COVID-19 pneumonitis.

**Methods:**

This multicentre, retrospective, cohort study using the Australian and New Zealand Intensive Care Society Adult Patient Database included patients aged ≥ 16 years admitted to 153 ICUs between 01/012020 and 12/31/2021 with admission diagnostic codes for viral pneumonia or acute respiratory distress syndrome, and Clinical Frailty Scale (CFS). The primary outcome was hospital mortality.

**Results:**

A total of 4620 patients were studied, and 3077 (66.6%) had COVID-19. The patients with COVID-19 were younger (median [IQR] 57.0 [44.7–68.3] vs. 66.1 [52.0–76.2]; *p* < 0.001) and less frail (median [IQR] CFS 3 [2–4] vs. 4 [3–5]; *p* < 0.001) than non-COVID-19 patients. The overall hospital mortality was similar between the patients with and without COVID-19 (14.7% vs. 14.9%; *p* = 0.82). Frailty alone as a predictor of mortality showed only moderate discrimination in differentiating survivors from those who died but was similar between patients with and without COVID-19 (AUROC 0.68 vs. 0.66; *p* = 0.42). Increasing frailty scores were associated with hospital mortality, after adjusting for Australian and New Zealand Risk of Death score and sex. However, the effect of frailty was similar in patients with and without COVID-19 (OR = 1.29; 95% CI: 1.19–1.41 vs. OR = 1.24; 95% CI: 1.11–1.37).

**Conclusion:**

The presence of frailty was an independent risk factor for mortality. However, the impact of frailty on outcomes was similar in COVID-19 patients compared to other causes of viral pneumonitis.

**Graphical Abstract:**

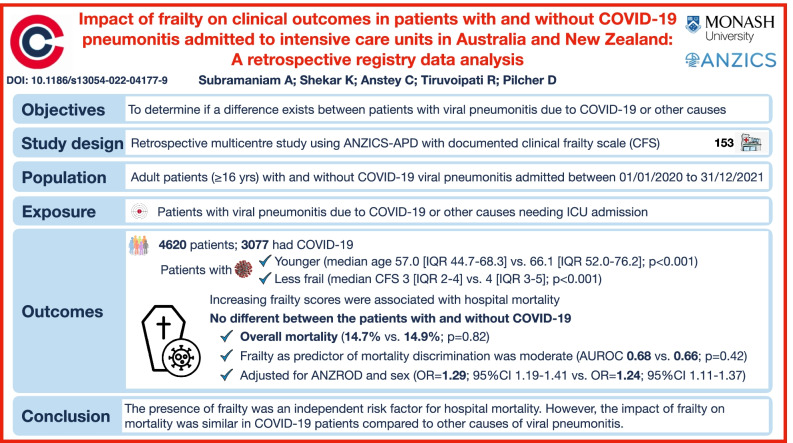

**Supplementary Information:**

The online version contains supplementary material available at 10.1186/s13054-022-04177-9.

## Introduction

The coronavirus disease 2019 (COVID-19) pandemic has had a devastating global impact. The clinical spectrum ranges widely from asymptomatic to severe respiratory failure, multiorgan failure, and death [1, 2]. Evidence suggests that older people with frailty are unequally affected [3], and that higher degrees of frailty along with cumulative comorbidities are linked with higher mortality in patients with COVID-19 [3–7].

Healthcare in many parts of the world has been severely strained due to insufficient intensive care unit (ICU) beds and workforce capacity [8]. This pressure resulted in triage systems to maximize efficient resource use [9–14]. Frailty assessment tools, such as the Clinical Frailty Scale (CFS), have been proposed as an adjunct to age-based criteria for critical care triage decisions (The National Institute for Health and Care Excellence, NICE triage guidelines) [15]. This guideline suggested that patients older than 65 years with a CFS score ≥ 5 might not benefit from ICU admission [10, 12, 16] and such patients were encouraged to establish goals of care documentation [9, 12, 13, 17]. Despite this, frail patients with COVID-19 were admitted to ICU and had greater mortality but spent relatively fewer days in ICU compared with non-frail patients [18].

Pre-pandemic, patients with frailty were also common among patients admitted to ICU. These patients had more than double the risk of death and functional dependence than patients without frailty [19–21]. A study in Australia and New Zealand found that a third of non-COVID-19 patients admitted to the ICU with pneumonia were frail and were associated with poor outcomes [22]. Frail patients with COVID-19 had a higher case fatality rate. It is, however, unclear if the impact of frailty on outcomes differs between patients with and without COVID-19. With the geographic isolation and very strict public health measures, the Australian and New Zealand healthcare system was not overwhelmed in 2020 [23], allowing improved access to ICU earlier to all patients, including those with frailty. This was, however, not the case in 2021 when a higher volume of cases put significant strain on the healthcare system in parts of Australia [24]. Consequently, we hypothesized that the presence of frailty in patients admitted to ICU with COVID-19 would be associated with worse outcomes than in patients with other “non-COVID-19” causes of viral pneumonitis. We aimed to determine whether the impact of frailty differed between patients with viral pneumonitis due to COVID-19 or other causes. Australia and New Zealand were uniquely placed to test this hypothesis because our health systems were not stretched as they were in other parts of the world and resource issues are less of a confounder.

## Methods

### Study design and setting

This was a retrospective multicentre cohort study, which analysed ICU admissions reported to the Australian and New Zealand Intensive Care Society (ANZICS) Adult Patient Database (APD) between 1 January 2020 and 31 December 2021.

### ANZICS-APD

The ANZICS-APD is a binational database that prospectively collects high-quality de-identified patient information, including demographics (such as age and sex), chronic health status, physiological and biochemical variables within the first 24 h of admission required for the Acute Physiology and Chronic Health Evaluation (APACHE)-III-j and IV illness severity scores and Australian and New Zealand Risk of Death (ANZROD), as well as ICU and hospital outcomes. Each patient is allocated a single diagnosis which reflects the primary cause of admission to ICU using the ANZICS modification of the APACHE-IV diagnosis coding system [25].

### Patient identification

Adult patients (age ≥ 16 years), with a documented CFS score, admitted to Australian and New Zealand ICUs with an ICU admission diagnosis of Viral Pneumonia or Acute Respiratory Distress Syndrome (ARDS) were included. Patients were further classified using a subcode of “suspected or confirmed pandemic infection” to indicate which were highly likely to have COVID-19 (Additional file 2: Table S1). Readmission episodes during the same hospitalization and admissions for organ donation or palliative care were excluded, as were patients with no primary outcome (hospital mortality) listed.

### Data extraction

Data included patient demographics (age, sex, comorbidities, ethnicity, ICU admission source, smoking status), obesity status (body mass index ≥ 30 kg/m^2^; data collected as patient’s height and weight in ANZICS-APD) CFS, ICU organ supports (need for mechanical ventilation, non-invasive ventilation, vasopressors, tracheostomy, extracorporeal membrane oxygenation [ECMO] and/or renal replacement therapies), treatment limitation order, the incidence of delirium (developed during the current ICU admission), ICU and hospital mortality, and respective length of stays. Treatment limitation was defined as that medical treatment would be constrained by either patient wishes or medical futility but does not necessarily imply that the patient was expected to die during this ICU admission.

### Frailty assessment

Frailty was measured with a modified version of the Canadian Study of Health and Aging Clinical Frailty Scale, which categorizes patients as non-frail (1 = very fit; 2 = well; 3 = managing well; 4 = vulnerable) or frail (5 = mild; 6 = moderate; 7 = severe; 8 = very severe) [26]. Terminally ill patients usually scored 9 on the CFS, and are instead scored in the ANZICS-APD on their level of frailty. This measurement has been validated among critically ill patients [19, 20] with good inter-rater reliability [19, 27, 28], and reported to be correlated with the other comprehensive frailty scales [29, 30]. In the ANZICS-APD, the CFS is modified to eight categories without a CFS-9 (terminally ill) [31]. The CFS represented the patient’s status in the two months preceding ICU admission [31]. For this study, we further grouped CFS scores according to five groups, CFS-1–3, CFS-4, CFS-5, CFS-6, and CFS-7–8 as reported in recent studies [32, 33]. The CFS was assigned by trained data collectors working in ICU, including junior doctors, nurses, and administrative staff, based on the patient’s level of physical function in the two months preceding ICU admission [25].

### Exposure and confounding variables

The exposure variable was frailty status based on CFS categories in patients with and without COVID-19. The confounding variables were illness severity (measured with ANZROD), and sex.

### Study aims and outcomes

We aimed to investigate whether the impact of frailty on mortality differed between patients with COVID-19 pneumonia and other aetiologies of viral pneumonia. The primary outcome was hospital mortality. Secondary outcomes included ICU mortality, ICU, and hospital length of stays and discharge destination.

### Subgroup analysis

Predefined subgroup analyses based on biological sex, age (≥ 65 years), and those needing mechanical ventilation were performed.

### Statistical analysis

The group comparisons between patients with and without COVID-19 were made using chi-square tests for proportions, student *t* tests for normally distributed data, and Mann–Whitney U or Kruskal–Wallis tests for nonparametric data depending on the number of categories examined. Data are reported as frequencies (%), means (standard deviations [SD]), or medians (interquartile range [IQR] 25–75%), respectively. Illness severity was determined using ANZROD, a highly discriminatory, locally derived, and well-calibrated mortality prediction model used for benchmarking ICU performance in Australia and New Zealand which combines age, chronic illnesses, acute physiological disturbance, and diagnosis [34, 35]. The association of CFS with hospital mortality in patients with and without COVID-19 was investigated using multivariable logistic regression with results reported as odds ratio (OR) and 95% confidence interval (CI). Model discrimination was assessed using the area under the receiver operating characteristic (AUROC) plots with the comparison between models assessed using chi-square tests [36]. Sensitivity subgroup analyses were performed which separately examined patients admitted in 2020 and 2021 (when there was a higher burden of COVID-19 admissions). Collinearity between ANZROD and CFS was assessed with variance inflation factor. Analyses were performed using SPSS software (version 27), and a two-sided *p* value of < 0.05 was used to indicate statistical significance.

## Results

During the study period, a total of 5735 patients were admitted to 153 Australian and New Zealand ICUs with admission diagnoses of either viral pneumonia or ARDS that were reported to the ANZICS-APD. Of these, 4620 patients had a documented CFS and were included in the study. There were no differences in age, sex, the proportion of treatment limitations, or hospitalization prior to ICU or pre-ICU hours between the 4620 patients and 1115 patients without a documented CFS. However, a lower proportion of those with missing frailty data had COVID-19 and these patients had higher illness severity scores (Additional file 2: Table S2).

Baseline characteristics are presented in Table 1. Patients with COVID-19 were younger (median age 57.0 [IQR 44.7–68.3] vs. 66.1 [IQR 52.0–76.2]; *p* < 0.001) and less frail (median CFS 3 [IQR 2–4] vs. 4 [IQR 3–5]; *p* < 0.001), than patients without COVID-19. A higher proportion of patients with COVID-19 were male (61.3% vs. 51.3%; *p* < 0.001). Patients with COVID-19 had lower APACHE-III scores and less frequently had chronic comorbidities such as respiratory, cardiovascular, renal, liver, immunosuppressive conditions, and metastatic cancer, but were more likely to be obese and have delirium, than patients without COVID-19. Admissions to ICU following a rapid response team review were less frequent for patients with COVID-19 (36.2% vs. 39.3%; *p* = 0.045). More patients with COVID-19 were receipt of mechanical ventilation, tracheostomies, ECMO therapies, and vasoactive agents, whereas fewer patients were receipt of non-invasive ventilation or renal replacement therapy than patients without COVID-19 (Additional file 1: Fig. S1). Further categorization by age and CFS categories is provided in Additional file 1: Fig. S2 and Additional file 2: Tables S3a and S3b.

**Table 1:** Baseline characteristics of patients with and without COVID-19

Variable	Patients with COVID-19	Patients without COVID-19	*p*-value
Number	3077	1543	–
Frailty status, *n* (%)			
CFS-1–3	2298 (74.7%)	620 (40.2%)	< 0.001
CFS-4	410 (13.3%)	408 (26.4%)	
CFS-5	157 (5.1%)	206 (13.4%)	
CFS-6	144 (4.7%)	203 (13.2%)	
CFS-7–8	68 (2.2%)	106 (6.9%)	
CFS—Frailty score (median [IQR])	3 (2, 4)	4 (3, 5)	< 0.001
Age (years) [median (IQR)]	57.0 (44.7, 68.3)	66.1 (52.0, 76.2)	< 0.001
Male sex, *n* (%)	1887 (61.3%)	792 (51.3%)	< 0.001
Indigenous status, *n* (%)	79 (2.7%)	139 (9.3%)	< 0.001
Jurisdiction, *n* (%)			
New South Wales	1486 (48.3%)	466 (30.2%)	< 0.001
Victoria	1387 (45.1%)	396 (25.7%)	
Queensland	37 (1.2%)	257 (16.7%)	
Western Australia	37 (1.2%)	117 (7.6%)	
South Australia	2 (0.1%)	62 (4.0%)	
Tasmania	2 (0.1%)	25 (1.6%)	
Australian Capital Territory	60 (1.9%)	62 (4.0%)	
Northern Territory	4 (0.1%)	49 (3.2%)	
New Zealand, *n* (%)	62 (2.0%)	109 (7.1%)	
Admission source, *n* (%)			
Home	2483 (80.7%)	1199 (77.7%)	< 0.001
Other acute hospital	280 (8.4%)	251 (16.3%)	
Nursing home or chronic care	15 (0.5%)	21 (1.4%)	
Other hospital ICU	260 (8.4%)	52 (3.4%)	
Rehabilitation	3 (0.1%)	6 (0.4%)	
Missing	36 (1.2%)	14 (0.9%)	
ICU admission source, *n* (%)			
Emergency department (ED)	1198 (38.9%)	660 (42.8%)	< 0.001
Ward	1486 (48.3%)	714 (46.3%)	
Other hospital (ED and ICU)	378 (12.2%)	161 (10.4%)	
Operating theatre/recovery	1 (0.0%)	2 (0.1%)	
Direct admit	14 (0.5%)	6 (0.4%)	
Documented co-morbidities, *n* (%)			
Chronic respiratory condition	201 (6.5%)	305 (19.8%)	< 0.001
Chronic cardiovascular condition	180 (5.8%)	189 (12.2%)	< 0.001
Chronic renal failure	74 (2.4%)	183 (11.9%)	< 0.001
Chronic liver disease	22 (0.7%)	37 (2.4%)	< 0.001
Diabetes mellitus	866 (29.3%)	415 (28.4%)	0.019
Immune suppressive therapy	147 (4.8%)	182 (11.8%)	< 0.001
Lymphoma	13 (0.4%)	29 (1.9%)	< 0.001
Leukaemia	26 (0.8%)	77 (5.0%)	< 0.001
Metastatic cancer	25 (0.8%)	59 (3.8%)	< 0.001
Obese (BMI ≥ 30 kg m^−2^)	1,061 (34.5%)	412 (26.7%)	< 0.001
Delirium	261 (8.5%)	116 (7.5%)	< 0.001
Pregnancy status	72 (2.3%)	13 (0.8%)	< 0.001
Pre-ICU (days) (median [IQR])	0.35 (0.13, 1.63)	0.38 (0.14, 1.39)	0.90
Organ failure scores			
APACHE III (mean [SD])	50.1 (20.0)	58.4 (21.7)	< 0.001
ANZROD (%) (mean [SD])	9.6 (12.3)	16.0 (18.2)	< 0.001
ICU admission post MET call	1107 (36.2%)	603 (39.3%)	0.045
Treatment limitations	248 (8.1%)	299 (19.4%)	< 0.001
Cardiac arrest, *n* (%)	6 (0.2%)	8 (0.5%)	0.08
ICU Supports			
Mechanical ventilation (MV), *n* (%)	1314 (43.2%)	328 (22.1%)	< 0.001
MV duration (hours), median (IQR)	178.0 (68.0, 348.8)	92.0 (37.0, 204.5)	< 0.001
Non-invasive ventilation (NIV), *n* (%)	1268 (41.9%)	750 (50.0%)	< 0.001
NIV duration (hours), median (IQR)	21.0 (4.3, 66.0)	11.0 (3.0, 30.0)	< 0.001
Vasopressor and inotropes, *n* (%)	1197 (39.3%)	461 (30.8%)	< 0.001
Renal replacement therapy, *n* (%)	182 (6.0%)	162 (10.9%)	< 0.001
Extracorporeal membrane oxygenation	106 (3.5%)	25 (1.7%)	< 0.001
Tracheostomy, *n* (%)	190 (6.3%)	38 (2.6%)	< 0.001

CFS: Clinical Frailty Scale, SD: standard deviation, IQR: interquartile range, BMI: body mass index, MET: medical emergency team, APACHE: Acute Physiology and Chronic Health Evaluation, ED: emergency department, ICU: intensive care unit, ROD: risk of death, ANZROD: Australia and New Zealand Risk of Death

Please refer to Additional file 2: Tables 3a and S3b for baseline characteristics based on CFS categories

### Primary outcome

**Fig. 1:**
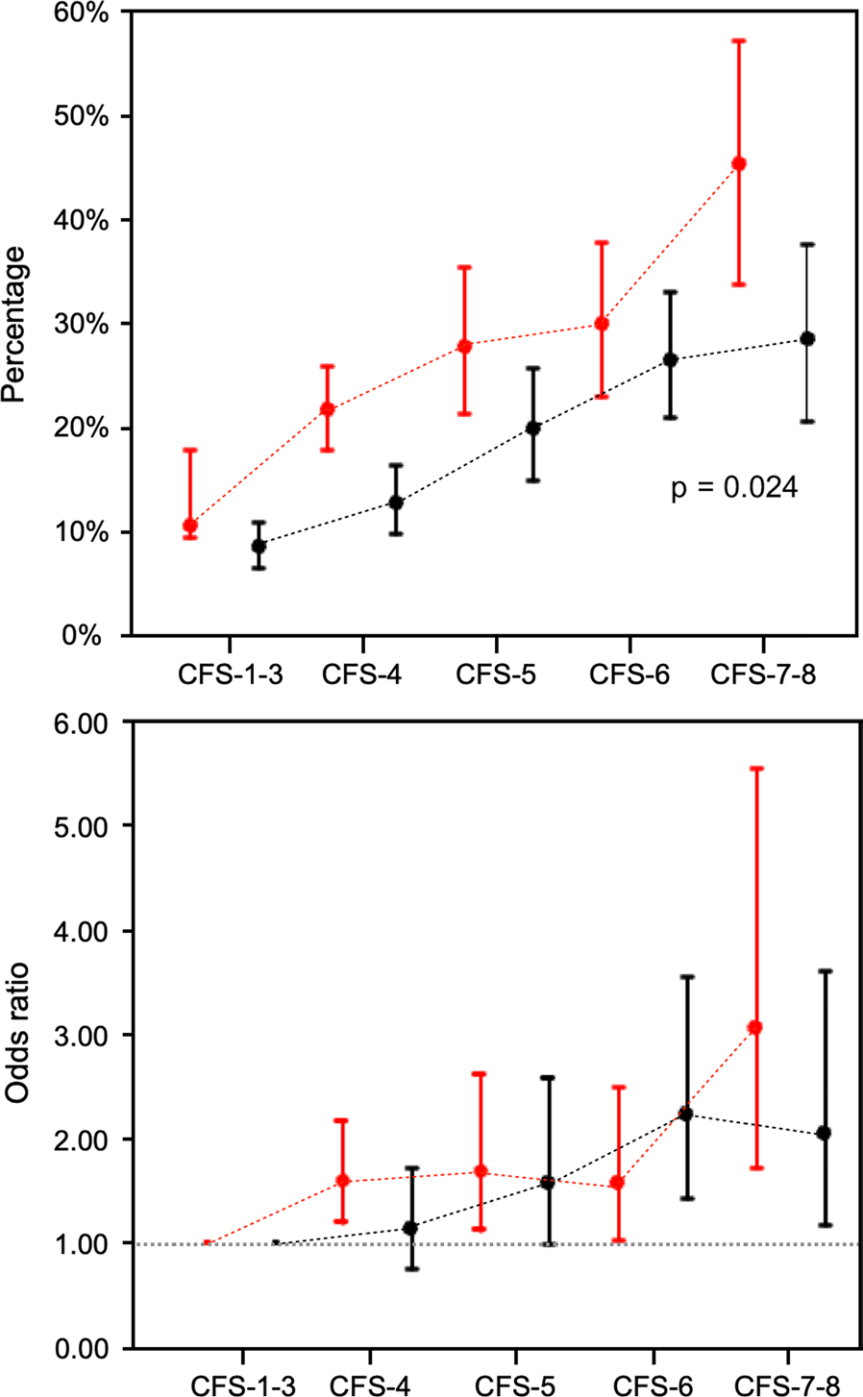
Hospital mortality according to Clinical Frailty Scale (CFS) score for all patients with (red lines) with and without (black lines) COVID-19. The top panel is unadjusted hospital mortality, while the bottom panel is adjusted for ANZROD and sex

Overall hospital mortality was similar between patients with and without COVID-19 (14.7% [441/3006] vs. 14.9% [280/1541]; *p* = 0.82). Higher hospital mortality was observed in COVID-19 patients compared to those without COVID-19 at equivalent frailty levels (*p* = 0.024; Fig. 1; Additional file 2: Table S4). Frailty was assessed using CFS as a continuous variable, increasing frailty scores were associated with mortality, after adjusting for ANZROD and sex. This effect was similar in patients with and without COVID-19 (OR = 1.29; 95% CI: 1.19–1.41 vs. OR = 1.24; 95% CI: 1.11–1.37; Table 2).

**Table 2:** Unadjusted hospital mortality in patients with and without COVID-19 (overall and at different levels of frailty) (Also refer to Fig. 2)

	Patients with COVID-19, OR (95% CI)	*p*-value	Patients without COVID-19, OR (95% CI)	*p*-value
*Frailty assessed using CFS as a continuous variable*				
CFS	1.29 (1.19–1.41)	< 0.001	1.24 (1.11–1.37)	< 0.001
Male sex	1.59 (1.25–2.04)	< 0.001	1.29 (0.94–1.77)	0.12
ANZROD	1.07 (1.06–1.08)	< 0.001	1.05 (1.04–1.06)	< 0.001

CFS: Clinical Frailty Scale; ANZROD: Australian and New Zealand Risk of Death

**Fig. 2:**
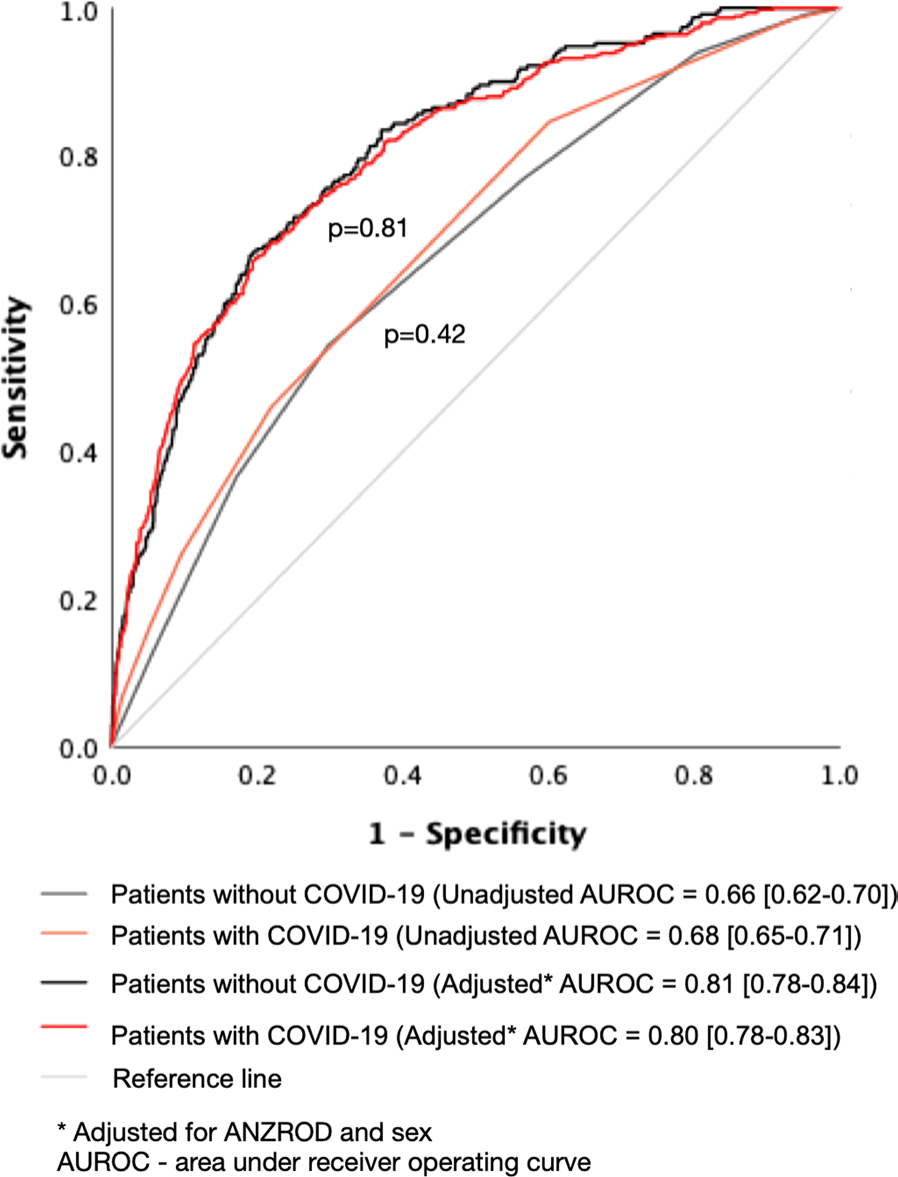
Area under the receiver operating curve with the Clinical Frailty Scale (CFS) treated as categories (CFS-1–3, CFS-4, CFS-5, CFS-6, and CFS-7–8). The comparison between models was assessed using chi-square tests and presented as p values

Frailty alone as a predictor of mortality showed only moderate discrimination in differentiating survivors from those who died. This effect was similar between patients with and without COVID-19 (AUROC 0.68 vs. 0.66; *p* = 0.42, Fig. 2). After adjusting for baseline illness severity (ANZROD) and sex, higher frailty scores were independently associated with mortality in patients with and without COVID-19. The presence of frailty (assessed as CFS categories) added little to the discriminatory capacity of the logistic regression model to predict death which already included ANZROD and sex (Fig. 1). The impact of frailty on mortality prediction was also no different between patients with and without COVID-19 (AUROC 0.80 vs. 0.81; *p* = 0.82; Fig. 2).

### Secondary outcomes

The unadjusted ICU mortality rates were higher only for non-frail patients with COVID-19 for CFS categories CFS-1–3 (8.6% vs. 5.8%; *p* = 0.023) and CFS-4 (17.1% vs. 8.9%; *p* < 0.001), compared to patients without COVID-19 (Additional file 2: Table S5). Patients with COVID-19 had a longer median length of stay in ICU than patients without COVID-19 (5.0 [IQR 2.1–10.9] vs. 3.0 [IQR 1.6–5.6] days; *p* < 0.001; Additional file 1: Fig. S3), especially for CFS categories CFS-1–3, CFS-4, and CFS-5. The median hospital length of stay was no different for patients with COVID-19 (12.9 [IQR 7.4–21.7] vs. 10.1 [IQR 5.4–18.8] days; *p* < 0.001) for CFS categories, than those without COVID-19 (*p* = 0.91). The ICU readmissions were lower in patients with COVID-19 for CFS categories CFS-1–3, CFS-4, and CFS-6, than in those without COVID-19 (*p* < 0.001). Overall, the patients with COVID-19 were less likely to be discharged home or to a nursing home, when compared to patients without COVID-19 (both *p* < 0.001) respectively.

### Subgroup analysis

#### Biological sex

**Fig. 3:**
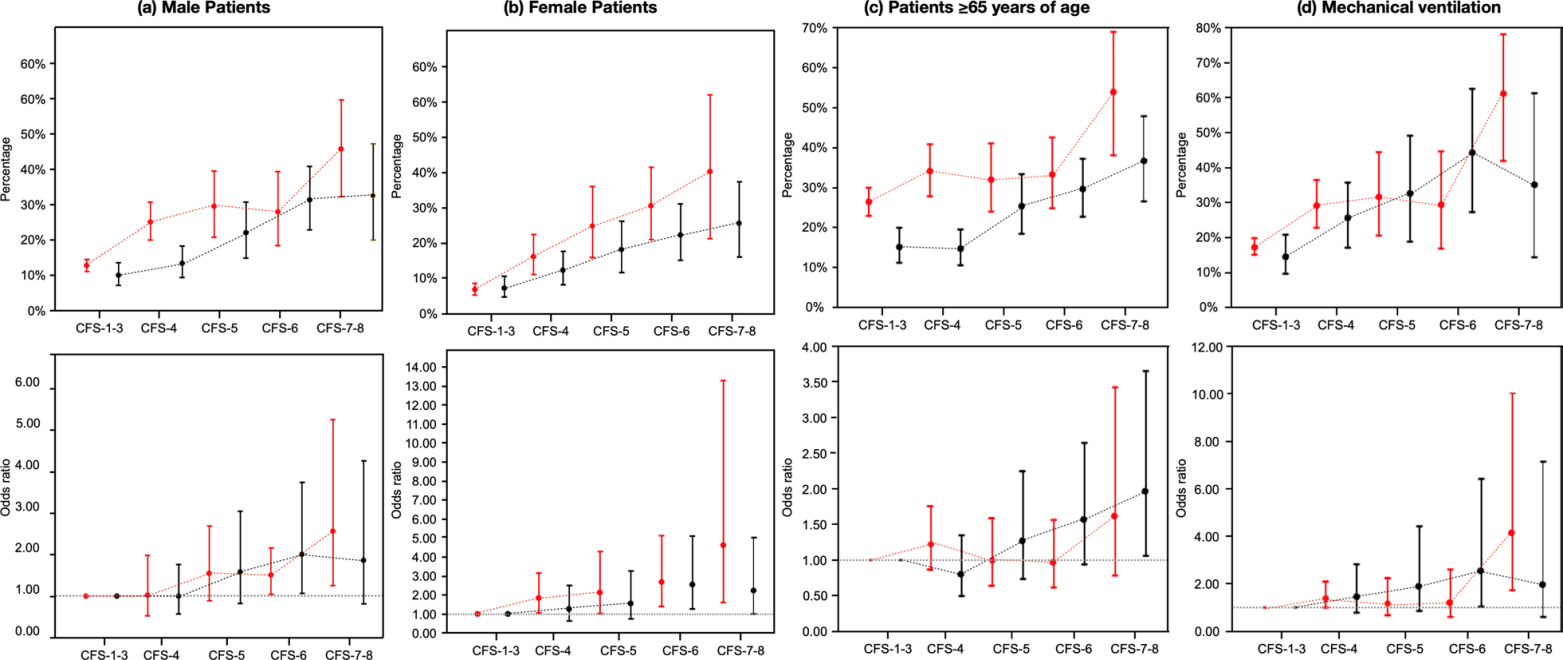
Hospital mortality according to Clinical Frailty Scale (CFS) categories for patients with (red) and without (black) COVID-19: **a** male sex, **b** female sex, **c** ≥ 65 years of age, and **d** those needing mechanical ventilation. The top panel is unadjusted hospital mortality, while the bottom panel is adjusted analysis. Biological sex was adjusted only for ANZROD, while others are adjusted for ANZROD and sex

More than half the patients were male (58.0%). A higher proportion of male patients had COVID-29 (70.4% vs. 61.3%; Additional file 2: Tables S5 and S6). The raw hospital mortality was higher for both male and female patients with COVID-19 for all CFS categories when compared to patients without COVID-19 (*p* < 0.001). Although the increasing frailty scores were associated with mortality, after adjusting for ANZROD, the effect of frailty was similar in both groups (Fig. 3; Additional file 2: Table S11).

#### Patients ≥ 65 years

Of the 1861, a lower proportion of patients with COVID-19 were ≥ 65 years, than those without COVID-19 (33.6% vs. 53.7%). Their median age was similar between the 2 groups (Additional file 2: Table S7). When compared to patients without COVID-19, the unadjusted hospital mortality was higher for patients with COVID-19 across all CFS categories, (*p* < 0.001). In patients aged ≥ 65 years, although the increasing frailty scores were associated with mortality, after adjusting for ANZROD and sex, the effect of frailty was similar in patients with and without COVID-19 (Fig. 3; Additional file 2: Table S11).

#### Patients needing mechanical ventilation

A total of 1642 patients (35.5%) were mechanically ventilated. Although more patients with COVID-19 received mechanical ventilation overall, (42.7% vs. 21.3%; Additional file 2: Table S8), the number of patients with frailty was lower than those without COVID-19 (9.5% vs. 24.1%). The raw mortality was higher for patients with COVID-19 for CFS categories CFS-1–3, CFS-4, and CFS-7–8, when compared to patients without COVID-19 (*p* < 0.001). Although the increasing frailty scores were associated with mortality, after adjusting for ANZROD and sex, the effect of frailty was similar in patients with and without COVID-19 (Fig. 3; Additional file 2: Table S11).

#### Patients admitted in 2020

Of the 1163 patients (25.2%) admitted, 38.1% (*n* = 444) had COVID-19 (Additional file 2: Table S9). The raw hospital mortality was higher for patients with COVID-19 for all CFS categories except CFS-7–8 when compared to patients without COVID-19 (*p* < 0.001). Despite the increasing frailty scores were associated with mortality, after adjusting for ANZROD and sex, the effect of frailty was similar in both groups (Additional file 1: Fig. S4; Additional file 2: Table S11).

#### Patients admitted in 2021

Of the 3457 patients (74.8%) admitted, 85.1% (*n* = 2942) were patients with COVID-19 (Additional file 2: Table S9). Of these, most were non-frail (75.2%, 2212/2942) when compared to patients without COVID-19 (69.4%, 675/972). The raw hospital mortality was higher for patients with COVID-19 for all CFS categories (*p* < 0.001). Although the increasing frailty scores were associated with mortality, after adjusting for ANZROD and sex, the effect of frailty was similar in patients with and without COVID-19 (Fig. 3; Additional file 2: Table S10).

## Discussion

### Summary of key findings

This multicentre retrospective observational study that compared viral pneumonia patients with and without COVID-19 admitted to ICU in Australia and New Zealand revealed that: firstly, the mortality increased with increasing frailty, but the impact of frailty was similar in patients with and without COVID-19 pneumonitis. Secondly, patients with COVID-19 were less frail and younger than patients without COVID-19. Thirdly, the CFS independently predicted hospital mortality in both patients with and without COVID-19 pneumonitis but had a low discriminatory capacity. Fourthly, only one in ten patients with frailty with COVID-19 received mechanical ventilation. Finally, the mortality was higher in patients ≥ 65 years of age and those requiring mechanical ventilation, especially with increasing frailty.

### Relationship to previous findings

There were few studies that compared outcomes of COVID-19 with influenza patients. These studies showed the overall hospital mortality; the need for ICU admission and mechanical ventilation was substantially higher in patients with COVID-19 [37–39]. However, none of these studies specifically investigated the association of frailty on clinically important outcomes. A recent study compared the characteristics and outcomes of very old patients with frailty with COVID-19 with historical controls and found that patients with COVID-19 were relatively less frail and had lower illness severity scores [40]. Our study was the first to compare critically ill patients with frailty, with and without COVID-19 pneumonitis, during the same period.

Our key study finding was that although the risk of death increased with frailty, the impact of frailty on hospital mortality was comparable between patients with and without COVID-19 pneumonitis. It is important to note that the Australian experience of the COVID-19 pandemic has differed from that internationally [41]. Our mortality rates were considerably lower than in other parts of the world. Importantly, we observed that the patients with COVID-19 were not only younger and less frail but also the overall proportion of patients with frailty was smaller when compared with those without COVID-19. Our study findings were, however, similar to a recent study that found that patients with COVID-19 were relatively less frail and had lower illness severity scores [40]. There was a higher proportion of patients with frailty in the non-COVID-19 group. Although outcomes in patients with frailty are bad [20, 22], the outcomes related to COVID-19 pneumonitis are worse. These two factors trade off against each other, explaining why the overall hospital mortality was similar between the two groups.

It is well established that higher degrees of frailty have been associated with poor outcomes and higher mortality rates during and after ICU admission [42, 43]. Similarly, we observed that hospital mortality increased with increasing frailty in both patients with and without COVID-19. A recent large prospective multinational study (COVIP) identified that frailty was independently associated with lower survival [11]. Our study found that although the CFS independently predicted hospital mortality, it had a low discriminatory capacity. Furthermore, the CFS was unable to clinically improve upon the predictability provided by baseline patient illness severity.

Patients with frailty were associated with lower use of mechanical ventilation [11, 44]. A recent systematic review observed that patients with frailty with COVID-19 were less commonly admitted to ICU or receive mechanical ventilation [45]. However, among those admitted to the ICU, almost two-thirds of patients with frailty with COVID-19 died in the hospital, with a greater risk of death for those receiving mechanical ventilation, when compared with patients without frailty [44]. Hospital mortality was relatively lower in these patients when compared to the published literature [11, 40, 44–46]. We observed that only 10% of patients with frailty with COVID-19 needed mechanical ventilation, which was lower than recently published in patients with COVID-19 [40, 45]. However, although the hospital mortality was higher, it was comparable in patients without COVID-19. This may indicate that frailty status was adopted as one of the triaging factors to screen patients for ICU admission and/or appropriate critical care interventions. These in turn may reinforce the importance of prudent selection and appropriate management of older patients with frailty amidst the pandemic, as previously observed [45].

Australia and New Zealand’s healthcare system was not as overwhelmed as other parts of the world in 2020. However, the impact was much greater in 2021. At the peak in October, 55% of all public ICU beds in Victoria and almost 40% of all public ICU beds in New South Wales were occupied by patients with COVID-19 [47]. This caused a significant strain in the healthcare system in late 2021 in both states, which was also associated with a deterioration in the risk-adjusted outcomes of COVID-19 patients [47, 48].

The inclusion period of 2 years encompassed patients from several different waves that also witnessed changes in treatment and the introduction of vaccinations. The rates of COVID-19 vaccine administration steadily increased since the rollout in February 2021. The vaccinations were initially prioritized in Victoria and New South Wales, which had the highest case numbers. At the end of the study period, > 90% of all eligible people had received the first dose Australia wide and > 84% received their second dose [49]. Further improvements in the outcomes in these patients are likely with increased uptake of vaccinations. However, despite vaccinations, public health measures to mitigate this pandemic, and new treatment options, COVID-19 may continue to severely impact frail older and vulnerable patients.

### Study implications

Our study found that frailty independently predicted mortality in both patients with and without COVID-19 pneumonitis, but the impact of frailty was similar in patients with and without COVID-19 pneumonitis. This implies that, regardless of COVID-19 status, the patients’ care was no different. This may at least in part reflect that Australia and New Zealand have evaded the magnitude of the pandemic that has overwhelmed healthcare systems in many parts of the world. Moreover, the intensive care resource availability is owing to stringent public health measures in Australia and New Zealand. Our study has demonstrated that more patients with COVID-19 admitted to ICU had CFS scores between 1 and 3. It is possible that the intensive care teams were more selective in admitting frail patients with COVID-19 into their ICUs.

### Strengths and limitations

Our study has several notable strengths. Firstly, the multicentre design, incorporating high-quality data Australia and New Zealand wide, as well as a larger sample size than many other studies. Secondly, the CFS, which is the most used frailty assessment tool for critically ill patients. Thirdly, we incorporated pre-specified several secondary analyses, to assess the impact of frailty on several important patient-centred ICU outcomes. To our knowledge, this is the only study to compare the impact of frailty among patients with and without COVID-19 pneumonitis. There are a few limitations to this study. Firstly, the retrospective study design meant that data collection was reliant on existing datasets and medical records. Secondly, despite the ANZICS-APD being recognized as a high-quality clinical registry dataset, the effect of data coding inaccuracy on the study findings could not be assessed. Thirdly, in the absence of having linkage to infection notification data and without site-based auditing of diagnostic coding over the pandemic, we cannot be certain about the degree of misclassification, if any. Fourthly, the CFS was adopted in the assessment of frailty in ICUs across Australia and New Zealand. Despite being an attractive tool to distinguish the different grades of frailty, the reliability of a single assessment tool may be inadequate, especially when it comes to justifying the rationing of medical treatment. Fifthly, the patients with COVID-19 admitted to the ICUs with an alternate diagnosis could have been missed. Sixthly, we do not have any information regarding the number of patients that were referred for ICU admission and denied ICU admission, or the COVID-19 pandemic may have led to an increased presentation of COVID-19 to the ICUs, while non-COVID-19 patients had a transient reduction in hospital presentations as well as ICU admissions. Seventhly, although some evidence suggests that CFS is a major determinant of long-term mortality in patients with COVID-19, we only reported on hospital mortality, as the ANZICS-APD only captured hospital-based outcomes. Finally, the Australia and New Zealand healthcare system has been very fortunate with the magnitude of COVID-19 infections being largely under control, and therefore, the results may not be generalizable in resource-constrained healthcare systems.

## Conclusion

This multicentre retrospective study that compared viral pneumonitis in patients with and without COVID-19 admitted to ICU in Australia and New Zealand found that patients with COVID-19 were younger and less frail than patients without COVID-19. The frailty independently predicted hospital mortality in both patients with and without COVID-19 pneumonitis but had low discriminatory capacity. The impact of frailty, however, was no different in patients with and without COVID-19.

## Supplementary Information

**Additional file 1: Figure S1** ICU Supports among patients with COVID-19 (red lines) with patients without COVID-19 (black lines), based on CFS score. Standard error bars are 95%-CI. **Figure S2** Age comparison based on CFS categories. The CFS categories are denoted by the different stacked colours starting with CFS 1–3 at the bottom up to CFS 7–8 at the top. **Figure S3** ICU bed days stratified by Clinical Frailty Scale (CFS) categories for patients with and without COVID-19. The bottom panels demonstrate the median length of stay among ICU survivors and non-survivors. **Figure S4** Hospital mortality according to Clinical Frailty Scale (CFS) categories for patients with (red) and without (black) COVID-19 for patients admitted in 2020 (a) and 2021 (b). The top panel is unadjusted hospital mortality, while the bottom panel is adjusted for ANZROD and sex.

**Additional file 2: Table S1** Diagnostic codes and subcodes for patients included in the study between 1st January 2020 and 31st December 2021. **Table S2** Missing data comparison for patients with and without the CFS scores. **Table S3a** CFS-1–3, CFS-4, and CFS-5 for patients with COVID-19 admitted to ICU with an admission diagnosis of viral pneumonia or ARDS. **Table S3b** CFS-6 and CFS-7–8 for patients with COVID-19 admitted to ICU with an admission diagnosis of viral pneumonia or ARDS. **Table S4** Comparison of unadjusted outcomes. Standard error bars are 95%-CI. **Table S5** Exposure and raw outcomes for male patients (2679 patients [58.0%]; with COVID-19 = 1887 patients; and without COVID-19 = 792 patients). **Table S6** Exposure and raw outcomes for female patients (1941 patients [42.0%]; with COVID-19 = 1190 patients; and without COVID-19 = 751 patients). **Table S7** Exposure and raw outcomes for patients ≥ 65 years (1861 patients [40.3%]; with COVID-19 = 1033 patients; and without COVID-19 = 828 patients). **Table S8** Exposure and raw outcomes in patients needing mechanical ventilation (1642 patients [35.5%]; COVID-19 = 1314 patients; non-COVID-19 = 328 patients). **Table S9** Exposure and raw outcomes for patients who were cared for in the year 2020 (1163 patients [25.2%]; with COVID-19 = 444 patients; and without COVID-19 = 719 patients). **Table S10** Exposure and raw outcomes for patients who were cared for in the year 2021 (3457 patients [74.8%]; with COVID-19 = 2942 patients; and without COVID-19 = 972 patients). **Table S11** Predictors for hospital mortality with the Clinical Frailty Scale (CFS) categories, adjusted for ANZROD and sex.

## Data Availability

The datasets generated and/or analysed during the current study are not publicly available as these are linked from three registries (ANZICS, VAED, and VDI), but are available from the corresponding author upon reasonable request.

## Abbreviations

ANZ: Australia and New Zealand
ANZICS: Australia and New Zealand Intensive Care Society
ANZROD: Australia and New Zealand Risk of Death
APACHE: Acute Physiology and Chronic Health Evaluation
APD: Adult Patient Database
AUROC: Area under the receiver operating characteristic
COPD: Chronic obstructive pulmonary disease
COVID-19: Coronavirus disease 2019
CFS: Clinical Frailty Scale
HR: Hazard ratio
ICU: Intensive care unit
IQR: Interquartile range
LOS: Length of stay
MI: Myocardial infarction
n: Number
OR: Odds ratio
RRT: Renal replacement therapy
SD: Standard deviation
