# Supplementary material for: Impact of frailty on clinical outcomes in patients with and without COVID-19 pneumonitis admitted to intensive care units in Australia and New Zealand: a retrospective registry data analysis
Source: Crit Care. 2022 Oct 3;26:301. doi: 10.1186/s13054-022-04177-9 (PMC9527725; doi:10.1186/s13054-022-04177-9)

**Impact of frailty on clinical outcomes in patients with and without COVID-19 pneumonitis admitted to intensive care units in Australia and New Zealand: A retrospective registry data analysis**

A/Prof Ashwin SUBRAMANIAM^1,2,3^ MBBS MMed FRACP FCICM

Prof Kiran SHEKAR^4, 5^, MBBS FCICM FCCCM PhD

A/Prof Christopher ANSTEY^6^ MBBS, MSc, FANZCA, FCICM

Prof Ravindranath Tiruvoipati^1,2^, MBBS, MS, MCh, FRCSEd, MSc, FCICM, EDIC, PhD

Prof David Pilcher ^3,7, 8^, MBBS MRCP(UK) FRACP FCICM

**Affiliations:**

1. Department of Intensive Care, Peninsula Health, Frankston, Victoria, Australia
2. Peninsula Clinical School, Monash University, Frankston, Victoria, Australia
3. Australian and New Zealand Intensive Care Research Centre (ANZIC-RC), School of Public Health and Preventive Medicine, Monash University, Melbourne, Victoria, Australia
4. Adult Intensive Care Services, The Prince Charles Hospital, Brisbane, Queensland, Australia
5. University of Queensland, Brisbane; Queensland University of Technology Brisbane and Bond University, Gold Coast, Queensland, Australia University of Queensland, Brisbane, Queensland, Australia,
6. Griffith University, Gold Coast, Queensland Australia
7. Department of Intensive Care, Alfred Hospital, Melbourne, Victoria, Australia
8. Centre for Outcome and Resource Evaluation, Australian and New Zealand Intensive Care Society, Melbourne, Victoria, Australia

**Legends**

**Supplementary Figure 1:** ICU Supports among patients with COVID-19 (red lines) with patients without COVID-19 (black lines), based on CFS score. Standard error bars are 95%-CI.

**Supplementary Figure 2:** Age comparison based on CFS categories. The CFS categories are denoted by the different stacked colours starting with CFS 1-3 at the bottom up to CFS 7-8 at the top.

**Supplementary Figure 3:** ICU bed days stratified by Clinical Frailty Scale (CFS) categories for patients with and without COVID-19. The bottom panels demonstrate the median length of stay among ICU survivors and non-survivors.

**Supplementary Figure 4:** Hospital mortality according to Clinical Frailty Scale (CFS) categories for patients with (red) and without (black) COVID-19 for patients admitted in 2020 (a) and 2021 (b). The top panel is unadjusted hospital mortality, while the bottom panel is adjusted for ANZROD and sex.

**Supplementary Figure 1:** ICU Supports among patients with COVID-19 (red lines) with patients without COVID-19 (black lines), based on CFS score. Standard error bars are 95%-CI.

|  |  |
| --- | --- |
|  |  |
|  |  |

**Supplementary Figure 2:** Age comparison based on CFS categories. The CFS categories are denoted by the different stacked colours starting with CFS 1-3 at the bottom up to CFS 7-8 at the top.


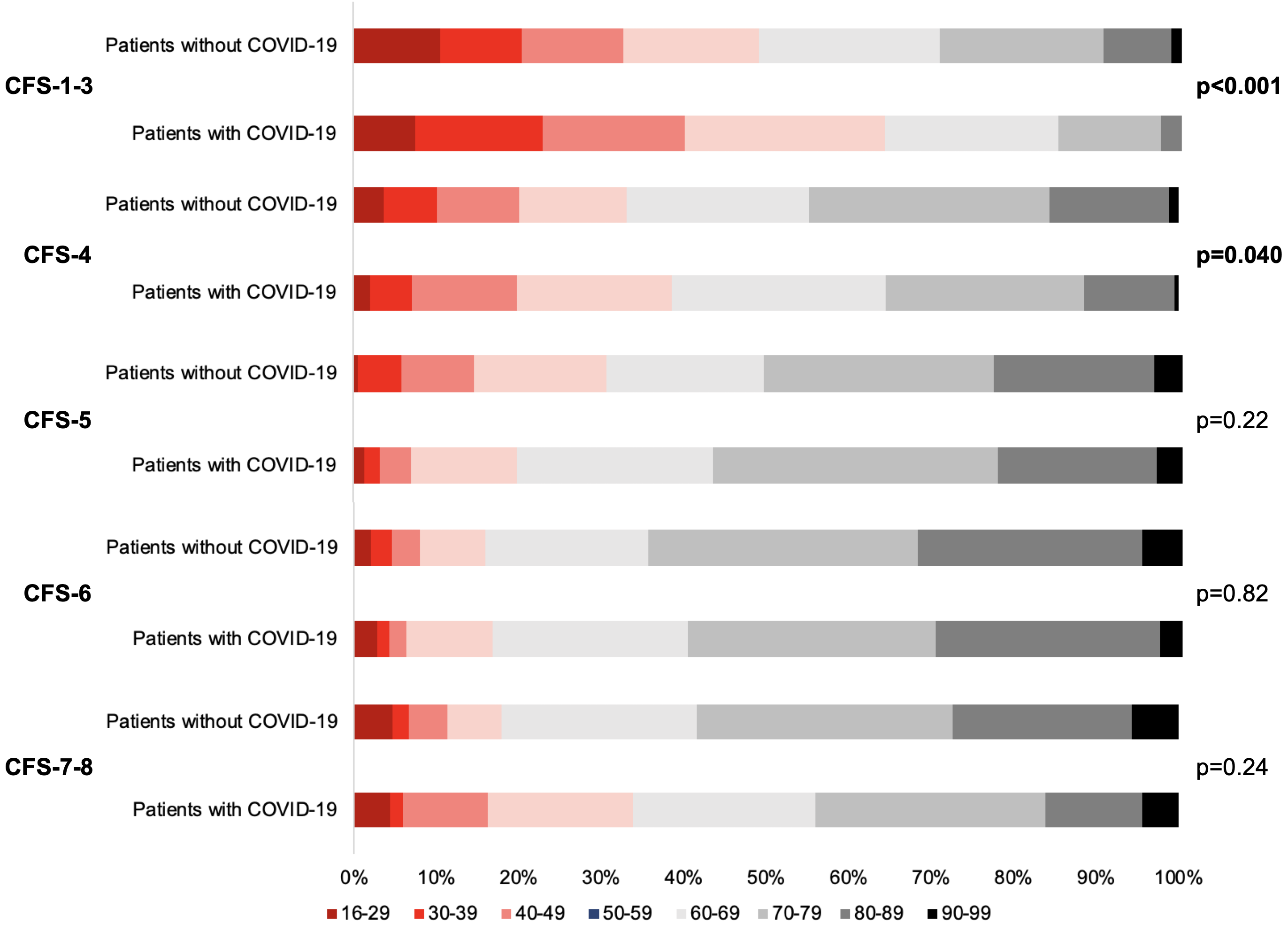


**Supplementary Figure 3:** ICU bed days stratified by Clinical Frailty Scale (CFS) categories for patients with and without COVID-19. The bottom panels demonstrate the median length of stay among ICU survivors and non-survivors.

| 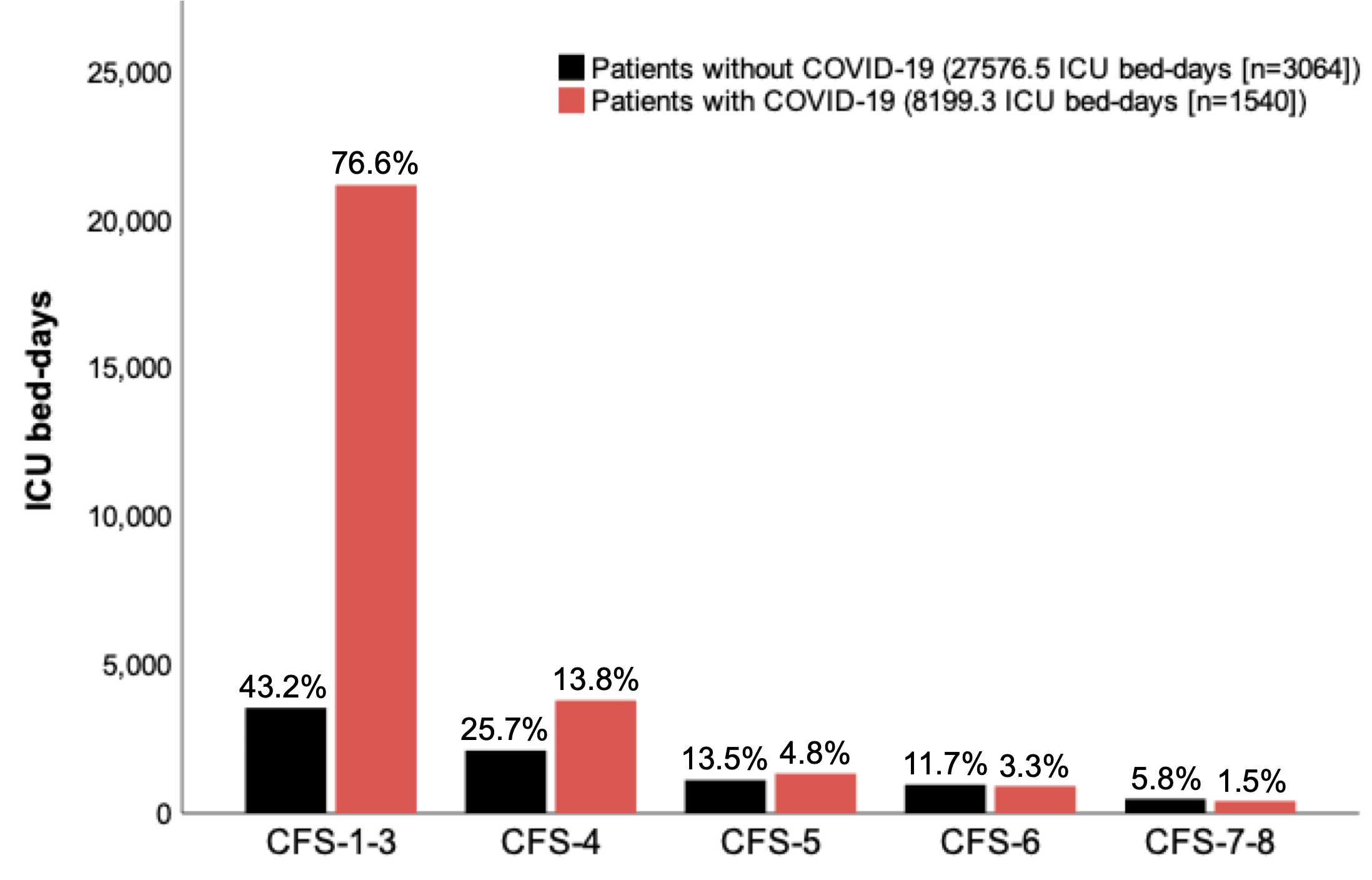 | 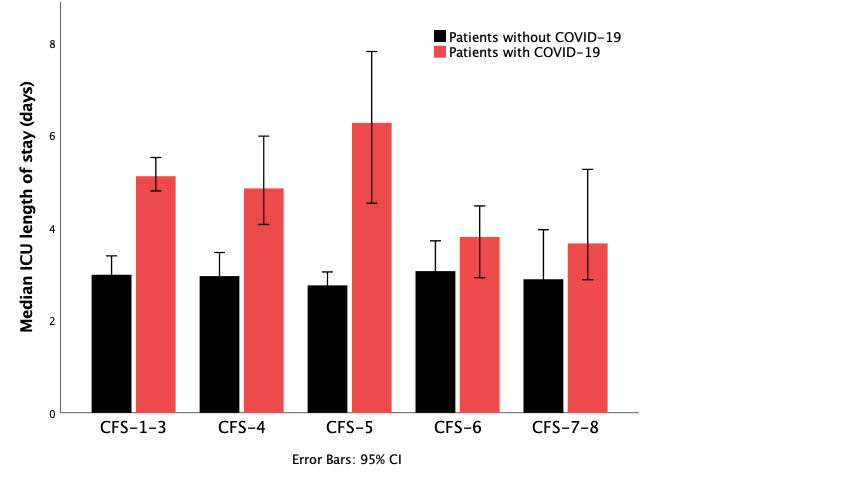 |
| --- | --- |
| 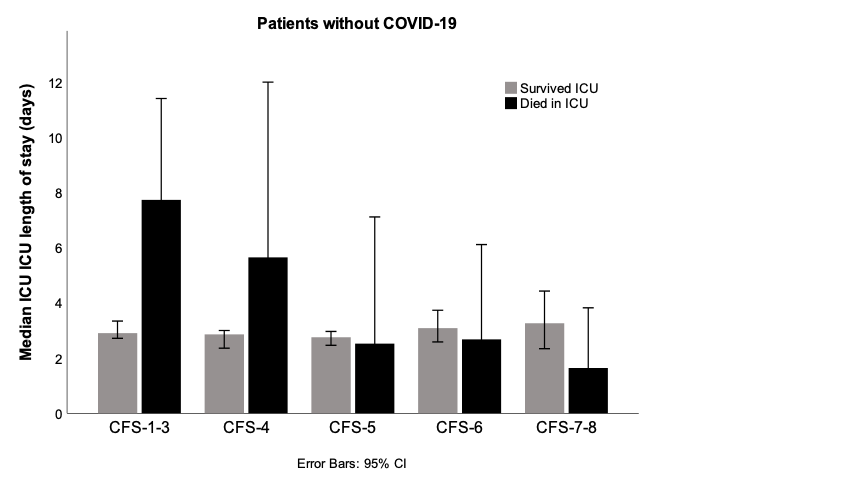 | 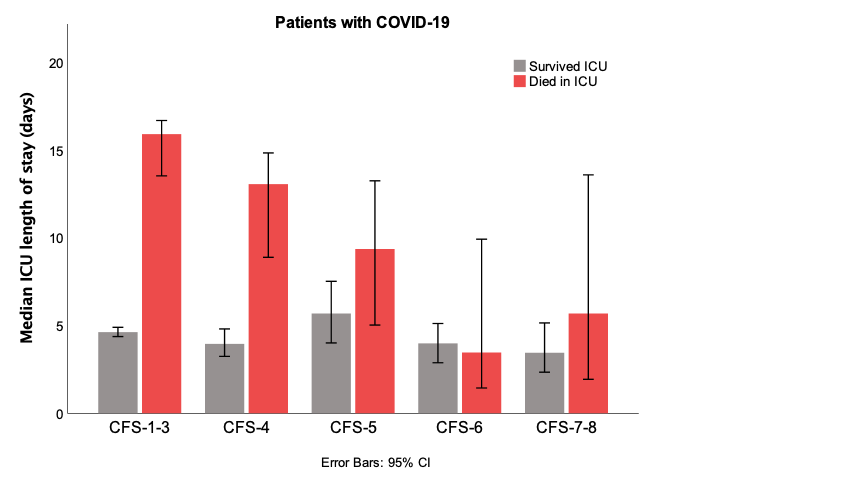 |

**Supplementary Figure 4:** Hospital mortality according to Clinical Frailty Scale (CFS) categories for patients with (red) and without (black) COVID-19 for patients admitted in 2020 (a) and 2021 (b). The top panel is unadjusted hospital mortality, while the bottom panel is adjusted for ANZROD and sex.


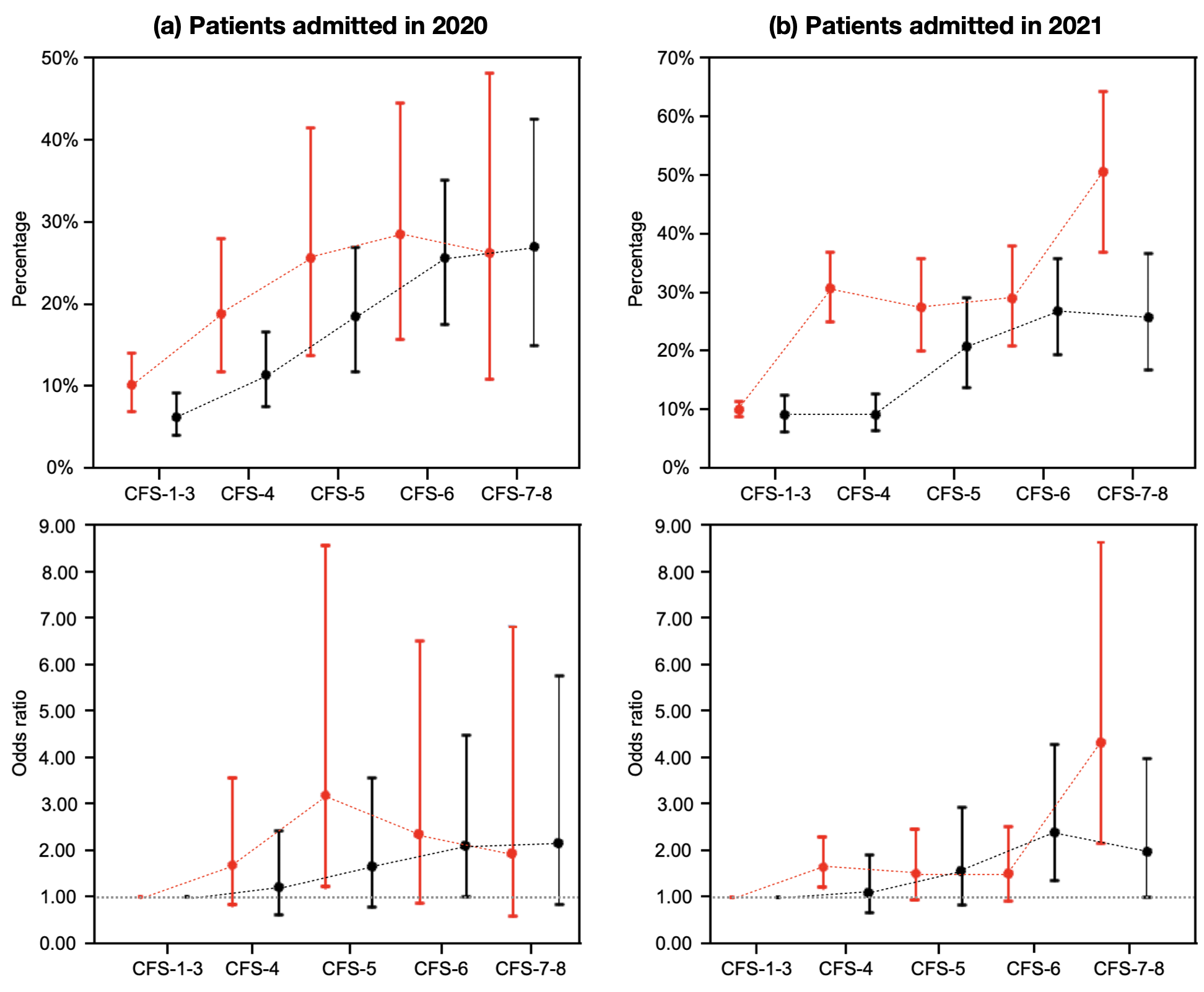

Supplement: Supplementary file 1 — Additional file 1: Figure S1 ICU Supports among patients with COVID-19 (red lines) with patients without COVID-19 (black lines), based on CFS score. Standard error bars are 95%-CI. Figure S2 Age comparison based on CFS categories. The CFS categories are denoted by the different stacked colours starting with CFS 1–3 at the bottom up to CFS 7–8 at the top. Figure S3 ICU bed days stratified by Clinical Frailty Scale (CFS) categories for patients with and without COVID-19. The bottom panels demonstrate the median length of stay among ICU survivors and non-survivors. Figure S4 Hospital mortality according to Clinical Frailty Scale (CFS) categories for patients with (red) and without (black) COVID-19 for patients admitted in 2020 (a) and 2021 (b). The top panel is unadjusted hospital mortality, while the bottom panel is adjusted for ANZROD and sex. [file 13054_2022_4177_MOESM1_ESM.docx]
