# Supplementary material for: Impact of frailty on clinical outcomes in patients with and without COVID-19 pneumonitis admitted to intensive care units in Australia and New Zealand: a retrospective registry data analysis
Source: Crit Care. 2022 Oct 3;26:301. doi: 10.1186/s13054-022-04177-9 (PMC9527725; doi:10.1186/s13054-022-04177-9)
Supplement: Supplementary file 2 — Additional file 2: Table S1 Diagnostic codes and subcodes for patients included in the study between 1st January 2020 and 31st December 2021. Table S2 Missing data comparison for patients with and without the CFS scores. Table S3a CFS-1–3, CFS-4, and CFS-5 for patients with COVID-19 admitted to ICU with an admission diagnosis of viral pneumonia or ARDS. Table S3b CFS-6 and CFS-7–8 for patients with COVID-19 admitted to ICU with an admission diagnosis of viral pneumonia or ARDS. Table S4 Comparison of unadjusted outcomes. Standard error bars are 95%-CI. Table S5 Exposure and raw outcomes for male patients (2679 patients [58.0%]; with COVID-19 = 1887 patients; and without COVID-19 = 792 patients). Table S6 Exposure and raw outcomes for female patients (1941 patients [42.0%]; with COVID-19 = 1190 patients; and without COVID-19 = 751 patients). Table S7 Exposure and raw outcomes for patients ≥ 65 years (1861 patients [40.3%]; with COVID-19 = 1033 patients; and without COVID-19 = 828 patients). Table S8 Exposure and raw outcomes in patients needing mechanical ventilation (1642 patients [35.5%]; COVID-19 = 1314 patients; non-COVID-19 = 328 patients). Table S9 Exposure and raw outcomes for patients who were cared for in the year 2020 (1163 patients [25.2%]; with COVID-19 = 444 patients; and without COVID-19 = 719 patients). Table S10 Exposure and raw outcomes for patients who were cared for in the year 2021 (3457 patients [74.8%]; with COVID-19 = 2942 patients; and without COVID-19 = 972 patients). Table S11 Predictors for hospital mortality with the Clinical Frailty Scale (CFS) categories, adjusted for ANZROD and sex. [file 13054_2022_4177_MOESM2_ESM.docx]

**Impact of frailty on clinical outcomes in patients with and without COVID-19 pneumonitis admitted to intensive care units in Australia and New Zealand: A retrospective registry data analysis**

A/Prof Ashwin SUBRAMANIAM^1,2,3^ MBBS MMed FRACP FCICM

Prof Kiran SHEKAR^4, 5^, MBBS FCICM FCCCM PhD

A/Prof Christopher ANSTEY^6^ MBBS, MSc, FANZCA, FCICM

Prof Ravindranath Tiruvoipati^1,2^, MBBS, MS, MCh, FRCSEd, MSc, FCICM, EDIC, PhD

Prof David Pilcher ^3,7, 8^, MBBS MRCP(UK) FRACP FCICM

**Affiliations:**

1. Department of Intensive Care, Peninsula Health, Frankston, Victoria, Australia
2. Peninsula Clinical School, Monash University, Frankston, Victoria, Australia
3. Australian and New Zealand Intensive Care Research Centre (ANZIC-RC), School of Public Health and Preventive Medicine, Monash University, Melbourne, Victoria, Australia
4. Adult Intensive Care Services, The Prince Charles Hospital, Brisbane, Queensland, Australia
5. University of Queensland, Brisbane; Queensland University of Technology Brisbane and Bond University, Gold Coast, Queensland, Australia University of Queensland, Brisbane, Queensland, Australia,
6. Griffith University, Gold Coast, Queensland Australia
7. Department of Intensive Care, Alfred Hospital, Melbourne, Victoria, Australia
8. Centre for Outcome and Resource Evaluation, Australian and New Zealand Intensive Care Society, Melbourne, Victoria, Australia

**Legends**

**Supplementary Table 1:** Diagnostic codes and subcodes for patients included in the study between 1^st^ January 2020 and 31^st^ December 2021.

**Supplementary Table 2:** Missing data comparison for patients with and without the CFS scores.

**Supplementary Table 3a:** CFS-1-3, CFS-4, and CFS-5 for patients with COVID-19 admitted to ICU with an admission diagnosis of viral pneumonia or ARDS.

**Supplementary Table 3b:** CFS-6 and CFS-7-8 for patients with COVID-19 admitted to ICU with an admission diagnosis of viral pneumonia or ARDS.

**Supplementary Table 4:** Comparison of unadjusted outcomes. Standard error bars are 95%-CI.

**Supplementary Table 5:** Exposure and raw outcomes for male patients (2,679 patients [58.0%]; with COVID-19=1,887 patients; and without COVID-19=792 patients).

**Supplementary Table 6:** Exposure and raw outcomes for female patients (1941 patients [42.0%]; with COVID-19=1,190 patients; and without COVID-19=751 patients).

**Supplementary Table 7:** Exposure and raw outcomes for patients ≥65 years (1,861 patients [40.3%]; with COVID-19=1,033 patients; and without COVID-19=828 patients).

**Supplementary Table 8:** Exposure and raw outcomes in patients needing mechanical ventilation (1642 patients [35.5%]; COVID-19=1,314 patients; non-COVID-19=328 patients).

**Supplementary Table 9:** Exposure and raw outcomes for patients who were cared for in the year 2020 (1,163 patients [25.2%]; with COVID-19=444 patients; and without COVID-19=719 patients).

**Supplementary Table 10:** Exposure and raw outcomes for patients who were cared for in the year 2021 (3,457 patients [74.8%]; with COVID-19=2,942 patients; and without COVID-19=972 patients).

**Supplementary Table 11:** Predictors for hospital mortality with the Clinical Frailty Scale (CFS) categories, adjusted for ANZROD and sex.

**Supplementary Table 1:** Diagnostic codes and subcodes for patients included in the study between 1^st^ January 2020 and 31^st^ December 2021.

| **Diagnosis** | **Diagnostic code in ANZICS-APD** | **Diagnostic subcode for patients without COVID19** | **Diagnostic subcode for patients with COVID-19*** |
| --- | --- | --- | --- |
| Viral pneumonia | 213 | 213.01 | 213.02 |
| ARDS | 204 | 204.01 | 204.22 |
| ANZICS – Australia New Zealand Intensive Care Society; APD – adult patient database; ARDS – acute respiratory distress syndrome  * The patients were considered highly likely to be positive for COVID-19 due to being coded as having associated ‘suspected or confirmed pandemic infection’. | | | |

**Supplementary Table 2:** Missing data comparison for patients with and without the CFS scores.

| **Variable** | **Frailty data present** | **Missing frailty data** | **p-value** |
| --- | --- | --- | --- |
| Number | 4,620 | 1,115 | - |
| - Australia, n (%) | 4,449 (96.3%) | 1,086 (97.4%) | 0.07 |
| - New Zealand, n. (%) | 171 (3.7%) | 29 (2.6%) |  |
| COVID-19 positive | 3,077 (66.6%) | 645 (57.8%) | <0.001 |
| Indigenous, n (%) | 218 (4.9%) | 50 (4.7%) | 0.77 |
| Male sex, n (%) | 2679 (58.0%) | 639 (57.3%) | 0.49 |
| Age (years) (median (IQR)) | 60.0 (46.31, 71.2) | 62.0 (50.0, 72.9) | 0.08 |
| **Hospital admission source, n (%)** |  | | |
| - Home | 3,682 (80.6%) | 900 (82.0%) | 0.32 |
| - Other acute hospital (not ICU/ED) | 229 (5.0%) | 53 (4.8%) |  |
| - Nursing home or chronic care | 36 (0.8%) | 15 (1.3%) |  |
| - Other hospital ICU | 312 (6.8%) | 60 (5.5%) |  |
| - Other hospital Emergency department | 302 (6.6%) | 68 (6.2%) |  |
| - Rehabilitation | 9 (0.2%) | 3 (0.3%) |  |
| **ICU admission source, n (%)** |  | | |
| - Emergency department | 1,858 (40.2%) | 488 (43.8%) | **<**0.001 |
| - Ward | 2,200 (40.2%) | 490 (43.9%) |  |
| - Other hospital | 213 (4.6%) | 61 (5.5%) |  |
| - Other hospital ICU | 326 (7.1%) | 58 (5.2%) |  |
| - Operating theatre / Recovery | 3 (0.1%) | 4 (0.4%) |  |
| - Direct admit | 20 (0.4%) | 14 (1.3%) |  |
| **Documented co-morbidities, n (%)** | | | |
| - Chronic respiratory condition | 506 (11.0%) | 93 (8.3%) | 0.010 |
| - Chronic cardiovascular condition | 369 (8.0%) | 40 (3.6%) | <0.001 |
| - Chronic renal failure | 257 (5.6%) | 48 (4.3%) | 0.09 |
| - Chronic liver disease | 59 (1.3%) | 19 (1.7%) | 0.27 |
| - Diabetes mellitus | 1,281 (27.7%) | 201 (18.0%) | <0.001 |
| - Immune suppressive therapy | 329 (7.1%) | 74 (6.6%) | 0.57 |
| - Lymphoma | 42 (0.9%) | 8 (0.7%) | 0.54 |
| - Leukaemia | 103 (2.2%) | 15 (1.3%) | 0.06 |
| - Metastatic cancer | 84 (1.8%) | 21 (1.9%) | 0.19 |
| - BMI ≥ 30 kg.m^-2^ | 1,473 (31.9%) | 346 (30.9%) | 0.69 |
| - Delirium, n (%) | 377 (8.2%) | 24 (2.2%) | <0.001 |
| **Organ failure score** |  | | |
| - APACHE III score (mean [SD]) | 52.9 (20.9) | 55.2 (22.8) | <0.001 |
| - ANZROD (%) (mean [SD]) | 11.7 (14.8) | 13.4 (15.7) | <0.001 |
| ICU admission post MET call, n (%) | 1,710 (37.3%) | 354 (32.1%) | 0.001 |
| Cardiac arrest, n (%) | 14 (0.3%) | 2 (0.2%) | 0.46 |
| Treatment limitation, n (%) | 547 (11.8%) | 160 (14.3%) | 0.11 |
| Pre-ICU (days) (median [IQR]) | 0.37 (0.13, 2.01) | 0.34 (0.14, 1.79) | 0.45 |
| CFS – clinical frailty scale, SD – standard deviation, IQR. – interquartile range, MET – medical emergency team, APACHE - Acute Physiology and Chronic Health Evaluation, ED – emergency department, ICU – intensive care unit, ROD – risk of death, ANZROD – Australia New Zealand risk of death  COVID-19 status was determined by the presence of the diagnostic sub-code “Suspected or confirmed pandemic infection.” | | | |

**Supplementary Table 3a:** CFS-1-3, CFS-4, and CFS-5 for patients with COVID-19 admitted to ICU with an admission diagnosis of viral pneumonia or ARDS.

| **Variable** | **CFS-1-3** | | | **CFS-4** | | | **CFS-5** | | |
| --- | --- | --- | --- | --- | --- | --- | --- | --- | --- |
|  | **Patients with COVID-19** | **Patients without COVID-19** | **p-value** | **Patients with COVID-19** | **Patients without COVID-19** | **p-value** | **Patients with COVID-19** | **Patients without COVID-19** | **p-value** |
| Number | 2,298 | 620 | - | 410 | 408 | - | 157 | 206 | - |
| Jurisdiction |  |  |  |  |  |  |  |  |  |
| - New South Wales | 1,111 (48.3%) | 196 (31.6%) | <0.001 | 183 (44.6%) | 105 (25.7%) | <0.001 | 75 (47.8%) | 52 (25.2%) | <0.001 |
| - Victoria | 1029 (44.8%) | 152 (24.5%) |  | 195 (47.6%) | 110 (27.0%) |  | 75 (47.8%) | 61 (29.6%) |  |
| - Queensland | 25 (1.1%) | 103 (16.6%) |  | 9 (2.2%) | 74 (18.1%) |  | 2 (1.3%) | 35 (17.0%) |  |
| - Western Australia | 24 (1.0%) | 43 (6.9%) |  | 10 (2.4%) | 38 (9.3%) |  | 1 (0.6%) | 15 (7.3%) |  |
| - South Australia | 2 (0.1%) | 20 (3.2%) |  | 0 (0) | 16 (3.9%) |  | 0 (0) | 12 (5.8%) |  |
| - Tasmania | 2 (0.1%) | 12 (1.9%) |  | 0 (0) | 8 (2.0%) |  | 0 (0) | 1 (0.5%) |  |
| - Australian Capital Territory | 48 (2.1%) | 21 (3.4%) |  | 5 (1.2%) | 15 (3.7%) |  | 4 (2.5%) | 13 (6.3%) |  |
| - Northern Territory | 4 (0.2%) | 14 (2.3%) |  | 0 (0) | 23 (5.6%) |  | 0 (0) | 9 (4.4%) |  |
| - New Zealand, n. (%) | 53 (2.3%) | 59 (9.5%) |  | 8 (2.0%) | 19 (4.7%) |  | 0 (0) | 8 (3.9%) |  |
| Male sex, n (%) | 1435 (62.4%) | 324 (52.3%) | <0.001 | 162 (39.5%) | 182 (44.6%) | 0.14 | 88 (56.1%) | 100 (48.5%) | 0.16 |
| Indigenous status, n (%) | 57 (2.6%) | 54 (9.0%) | <0.001 | 17 (4.3%) | 50 (12.6%) | <0.001 | 0 (0) | 20 (10.0%) | <0.001 |
| Age (years), median (IQR) | 53.5 (40.9, 64.6) | 59.6 (44.1, 71.3) | <0.001 | 63.6 (52.4, 73.6) | 67.0 (53.2, 76.8) | 0.040 | 71.7 (63.0, 78.1) | 69.7 (57.2, 79.3) | 0.21 |
| **Admission source, n (%)** |  |  |  |  |  |  |  |  |  |
| - Home | 1,851 (80.5%) | 469 (75.6%) | <0.001 | 349 (85.1%) | 329 (80.6%) | 0.29 | 121 (77.1%) | 158 (76.7%) | 0.12 |
| - Other acute hospital (not ICU/ED) | 13 (3.2%) | 47 (6.6%) |  | 2 (2.4%) | 11 (5.2%) |  | 7 (4.5%) | 17 (8.3%) |  |
| - Rehabilitation | 1 (0.0%) | 1 (0.2%) |  | 1 (0.2%) | 1 (0.2%) |  | 0 (0) | 0 (0) |  |
| - Other hospital ICU | 206 (9.0%) | 26 (4.2%) |  | 28 (6.8%) | 13 (3.2%) |  | 15 (5.7%) | 9 (4.4%) |  |
| - Other hospital ED | 114 (5.0%) | 69 (11.1%) |  | 15 (3.7%) | 33 (8.1%) |  | 9 (5.7%) | 19 (9.2%) |  |
| - Nursing home | 0 (0) | 0 (0) |  | 0 (0) | 0 (0) |  | 4 (2.5%) | 3 (1.5%) |  |
| - Missing | 1 (0.0%) | 2 (0.3%) |  | 3 (0.7%) | 4 (1.0%) |  | 1 (0.6%) | 0 (0) |  |
| **ICU admission source, n (%)** |  |  |  |  |  |  |  |  |  |
| - ED | 889 (38.7%) | 273 (44.0%) | <0.001 | 162 (41.7%) | 170 (41.7%) | 0.35 | 51 (32.5%) | 87 (42.2%) | 0.023 |
| - Ward | 1,100 (47.9%) | 267 (43.1%) |  | 210 (51.2%) | 202 (49.5%) |  | 88 (56.1%) | 96 (46.6%) |  |
| - Other hospital | 73 (3.2%) | 53 (8.5%) |  | 13 (3.2%) | 19 (4.7%) |  | 5 (3.2%) | 16 (7.8%) |  |
| - Other hospital ICU | 223 (9.7%) | 26 (4.1%) |  | 25 (6.1%) | 16 (3.9%) |  | 13 (8.2%) | 6 (2.9%) |  |
| - Operating theatre | 1 (0.0%) | 0 (0) |  | 0 (0) | 1 (0.2%) |  | 0 (0) | 0 (0) |  |
| - Direct admit | 12 (0.5%) | 1 (0.2%) |  | 0 (0) | 0 (0) |  | 0 (0) | 1 (0.5%) |  |
| **Documented co-morbidities, n (%)** | | | | | | | | | |
| - Chronic respiratory condition | 83 (3.6%) | 61 (9.8%) | 0.001 | 38 (9.3%) | 81 (19.9%) | <0.001 | 23 (14.6%) | 48 (23.3%) | 0.040 |
| - Chronic cardiovascular condition | 70 (3.0%) | 44 (7.1%) | 0.001 | 41 (10.0%) | 60 (14.7%) | 0.041 | 25 (15.9%) | 30 (14.6%) | 0.72 |
| - Chronic renal failure | 18 (0.8%) | 24 (7.3%) | <0.001 | 26 (6.3%) | 61 (15.0%) | 0.029 | 12 (7.6%) | 38 (18.4%) | 0.003 |
| - Chronic liver disease | 7 (0.3%) | 7 (1.1%) | 0.008 | 5 (1.2%) | 10 (2.5%) | 0.19 | 4 (2.5%) | 9 (4.4%) | 0.36 |
| - Diabetes mellitus | 551 (25.1%) | 113 (19.6%) | 0.014 | 165 (41.3%) | 124 (29.8%) | 0.014 | 63 (41.2%) | 71 (36.4%) | 0.29 |
| - Immune suppressive therapy | 66 (2.9%) | 55 (8.9%) | <0.001 | 39 (9.5%) | 59 (14.5%) | 0.029 | 19 (12.1%) | 30 (14.6%) | 0.50 |
| - Lymphoma | 6 (0.3%) | 9 (1.5%) | <0.001 | 2 (0.5%) | 10 (2.5%) | 0.020 | 2 (1.3%) | 4 (1.9%) | 0.62 |
| - Leukaemia | 8 (0.3%) | 25 (4.0%) | <0.001 | 7 (1.7%) | 33 (8.1%) | <0.001 | 5 (3.2%) | 10 (4.9%) | 0.43 |
| - Metastatic cancer | 7 (0.3%) | 14 (2.3%) | <0.001 | 6 (1.5%) | 9 (2.2%) | 0.43 | 4 (2.5%) | 12 (5.8%) | 0.13 |
| - Delirium, n (%) | 163 (7.1%) | 42 (6.8%) | <0.001 | 47 (11.5%) | 28 (6.9%) | <0.001 | 21 (13.4%) | 21 (10.2%) | 0.08 |
| - Obese (BMI ≥ 30 kg.m^-2^) | 795 (34.6%) | 164 (26.5%) | <0.001 | 102 (24.9%) | 149 (36.5%) | <0.001 | 56 (35.7%) | 61 (29.6%) | 0.09 |
| Pregnancy status | 70 (3.0%) | 12 (1.9%) | 0.33 | 2 (0.5%) | 1 (0.2%) | 0.56 | 0 (0) | 0 (0) | - |
| Pre-ICU (Hours) (median [IQR]) | 11.2 (5.3, 91.7) | 8.1 (3.0, 37.1) | 0.90 | 11.2 (5.3, 91.7) | 10.5 (3.3, 74.0) | 0.29 | 8.6 (2.6, 22.1) | 12.9 (4.8, 76.4) | 0.08 |
| **Organ failure scores** |  |  |  |  |  |  |  |  |  |
| - APACHE 3 (mean [SD]) | 47.0 (18.6) | 52.0 (21.3) | <0.001 | 56.0 (19.4) | 60.1 (19.5) | 0.90 | 61.4 (20.7) | 64.7 (21.8) | 0.24 |
| - ANZROD (%) (mean [SD]) | 7.3 (9.2) | 10.4 (13.3) | <0.001 | 13.0 (14.2) | 16.0 (17.7) | <0.001 | 18.1 (18.1) | 20.6 (20.9) | 0.009 |
| ICU admission post MET call | 811 (35.5%) | 214 (34.8%) | 0.73 | 156 (38.5%) | 174 (42.9%) | 0.21 | 67 (42.9%) | 85 (41.5%) | 0.78 |
| Cardiac arrest, n (%) | 3 (0.1%) | 3 (0.5%) | 0.10 | 1 (0.3%) | 1 (0.2%) | 0.98 | 0 (0) | 1 (0.5%) | 0.39 |
| Treatment limitation | 61 (2.7%) | 43 (6.9%) | <0.001 | 55 (13.4%) | 70 (17.2%) | 0.33 | 39 (24.8%) | 52 (25.2%) | 0.58 |
| **ICU Supports provided** |  |  |  |  |  |  |  |  |  |
| - Mechanical ventilation (MV) | 1,015 (44.6%) | 162 (27.3%) | <0.001 | 174 (43.1%) | 87 (22.1%) | <0.001 | 59 (38.1%) | 36 (18.2%) | <0.001 |
| - MV duration (hours) | 178 (69, 352) | 102 (29, 228) | <0.001 | 189 (61, 354) | 88 (44, 235) | 0.013 | 193 (43, 294) | 87 (47, 154) | 0.33 |
| - Non-invasive ventilation (NIV) | 914 (40.3%) | 255 (42.6%) | 0.31 | 183 (45.4%) | 214 (53.8%) | 0.018 | 73 (47.7%) | 105 (53.0%) | 0.32 |
| - NIV duration (hours) | 22 (4, 64) | 11 (3, 32) | <0.001 | 18 (4, 69) | 9 (3, 26) | <0.001 | 37(5, 105) | 9 (3, 25) | <0.001 |
| - Inotropes | 883 (38.8%) | 182 (30.4%) | <0.001 | 178 (44.1%) | 125 (31.6%) | <0.001 | 57 (36.8%) | 61 (30.5%) | 0.21 |
| - Renal replacement therapy | 126 (5.6%) | 40 (6.7%) | 0.28 | 35 (8.7%) | 71 (18.0%) | <0.001 | 8 (5.2%) | 34 (17.3%) | <0.001 |
| - ECMO | 93 (4.1%) | 15 (2.5%) | 0.07 | 11 (2.7%) | 7 (1.8%) | 0.36 | 1 (0.7%) | 3 (1.5%) | 0.45 |
| - Tracheostomy | 151 (6.7%) | 20 (3.4%) | 0.06 | 25 (6.2%) | 10 (2.5%) | 0.011 | 7 (4.6%) | 4 (2.0%) | 0.18 |
| CFS – clinical frailty scale, SD – standard deviation, IQR. – interquartile range, BMI – body mass index, MET – medical emergency team, APACHE - Acute Physiology and Chronic Health Evaluation, ED – emergency department, ICU – intensive care unit, ROD – risk of death, ANZROD – Australia New Zealand risk of death | | | | | | | | | |

**Supplementary Table 3b:** CFS-6 and CFS-7-8 for patients with COVID-19 admitted to ICU with an admission diagnosis of viral pneumonia or ARDS. Data are summarized according to distribution if normal.

|  | **CFS-6** | | | **CFS-7-8** | | |
| --- | --- | --- | --- | --- | --- | --- |
| **Variable** | **Patients with COVID-19** | **Patients without COVID-19** | **p-value** | **Patients with COVID-19** | **Patients without COVID-19** | **p-value** |
| Number | 144 | 203 |  | 68 | 106 |  |
| - New South Wales | 80 (55.6%) | 78 (38.4%) | <0.001 | 37 (54.4%) | 35 (33.0%) | <0.001 |
| - Victoria | 57 (39.6%) | 44 (21.7%) |  | 31 (45.6%) | 29 (27.4%) |  |
| - Queensland | 1 (0.7%) | 31 (15.3%) |  | 0 (0) | 14 (13.2%) |  |
| - Western Australia | 2 (1.4%) | 15 (7.4%) |  | 0 (0) | 6 (5.7%) |  |
| - South Australia | 0 (0) | 7 (3.4%) |  | 0 (0) | 7 (6.6%) |  |
| - Tasmania | 0 (0) | 2 (1.0%) |  | 0 (0) | 2 (1.9%) |  |
| - Australian Capital Territory | 3 (2.1%) | 11 (5.4%) |  | 0 (0) | 2 (1.9%) |  |
| - Northern Territory | 0 (0) | 2 (1.0%) |  | 0 (0) | 1 (0.9%) |  |
| - New Zealand, n. (%) | 1 (0.7%) | 13 (6.4%) |  | 0 (0) | 10 (9.4%) |  |
| Male sex | 68 (47.2%) | 99 (48.8%) | 0.78 | 48 (70.6%) | 43 (40.6%) | <0.001 |
| Indigenous status | 1 (0.7%) | 8 (4.0%) | 0.07 | 4 (6.3%) | 7 (6.7%) | 0.92 |
| Age (years) | 72.0 (63.9, 81.7) | 73.2 (63.4, 81.8) | 0.50 | 70.8 (58.5, 79.6) | 71.6 (64.7, 79.5) | 0.031 |
| **Admission source, n (%)** |  |  |  |  |  |  |
| - Home | 114 (79.2%) | 168 (82.8%) | 0.14 | 48 (70.6%) | 75 (70.8%) | 0.29 |
| - Other acute hospital (not ICU/ED) | 9 (6.3%) | 7 (3.4%) |  | 7 (10.3%) | 4 (3.8%) |  |
| - Rehabilitation | 0 (0) | 2 (1.0%) |  | 1 (1.5%) | 2 (1.9%) |  |
| - Other hospital ICU | 7 (4.9%) | 2 (1.0%) |  | 4 (5.9%) | 2 (1.9%) |  |
| - Other hospital ED | 9 (6.3%) | 18 (8.9%) |  | 4 (5.9%) | 12 (11.3%) |  |
| - Nursing home | 5 (3.5%) | 5 (2.5%) |  | 4 (5.9%) | 10 (9.4%) |  |
| - Missing | 0 (0) | 1 (0.5%) |  | 0 (0) | 1 (0.9%) |  |
| **ICU admission source, n (%)** |  |  |  |  |  |  |
| - ED | 70 (48.6%) | 85 (41.9%) | 0.05 | 27 (39.7%) | 45 (42.5%) | 0.08 |
| - Ward | 59 (41.0%) | 95 (46.8%) |  | 30 (44.1%) | 54 (50.9%) |  |
| - Other hospital | 6 (4.2%) | 17 (8.4%) |  | 6 (8.8%) | 5 (4.7%) |  |
| - Other hospital ICU | 7 (4.9%) | 2 (1.0%) |  | 4 (5.9%) | 2 (1.9%) |  |
| - Operating theatre / Recovery | 0 (0) | 1 (0.9%) |  | 0 (0) | 0 (0) |  |
| - Direct admit | 1 (0.7%) | 3 (1.5%) |  | 1 (1.5%) | 1 (0.9%) |  |
| **Documented co-morbidities, n (%)** |  |  |  |  |  |  |
| - Chronic respiratory condition | 35 (24.3%) | 76 (37.4%) | 0.010 | 22 (32.4%) | 39 (36.8%) | 0.55 |
| - Chronic cardiovascular condition | 27 (18.8%) | 40 (19.7%) | 0.82 | 17 (25.0%) | 23 (21.7%) | 0.61 |
| - Chronic renal failure | 14 (9.4%) | 29 (14.3%) | 0.20 | 4 (5.9%) | 11 (10.4%) | 0.30 |
| - Chronic liver disease | 3 (2.1%) | 7 (3.4%) | 0.45 | 3 (4.4%) | 4 (3.8%) | 0.83 |
| - Diabetes mellitus | 64 (46.0%) | 70 (35.7%) | 0.26 | 23 (34.3%) | 37 (36.7%) | 0.42 |
| - Immune suppressive therapy | 16 (11.1%) | 26 (12.8%) | 0.63 | 7 (10.3%) | 12 (11.3%) | 0.83 |
| - Lymphoma | 2 (1.4%) | 5 (2.5%) | 0.48 | 1 (1.5%) | 1 (0.9%) | 0.75 |
| - Leukaemia | 3 (2.1%) | 6 (3.0%) | 0.61 | 3 (4.4%) | 3 (2.8%) | 0.58 |
| - Metastatic cancer | 5 (3.5%) | 14 (6.9%) | 0.17 | 3 (4.4%) | 10 (9.4%) | 0.22 |
| - Delirium | 20 (13.9%) | 17 (8.4%) | 0.07 | 10 (14.7%) | 8 (7.5%) | 0.21 |
| - Obese (BMI ≥ 30 kg.m^-2^) | 31 (21.5%) | 48 (23.6%) | 0.22 | 27 (39.7%) | 30 (28.3%) | 0.26 |
| Pregnancy status | 0 (0) | 0 (0) | - | 0 (0) | 0 (0) | - |
| Pre-ICU (days) | 5.9 (3.3, 11.2) | 7.6 (0.7, 74.5) | 0.39 | 8.1 (2.3, 70.6) | 11.5 (3.6, 101.8) | 0.31 |
| **Organ failure scores** |  |  |  |  |  |  |
| - APACHE 3 (mean [SD]) | 63.8 (20.3) | 64.0 (21.1) | 0.14 | 63.6 (24.4) | 66.3 (21.9) | 0.50 |
| - ANZROD (%) (mean [SD]) | 20.6 (17.5) | 22.6 (20.9) | 0.013 | 23.2 (22.7) | 26.8 (22.2) | 0.78 |
| ICU admission post MET call | 94 (34.3%) | 82 (40.4%) | 0.25 | 24 (35.3%) | 48 (45.3%) | 0.19 |
| Cardiac arrest | 0 (0) | 2 (1.0%) | 0.25 | 2 (3.2%) | 1 (1.0%) | 0.29 |
| Treatment limitation | 64 (44.4%) | 79 (38.9%) | 0.53 | 29 (42.6%) | 55 (51.9%) | 0.49 |
| **ICU Supports provided** |  |  |  |  |  |  |
| - Mechanical ventilation (MV) | 40 (28.6%) | 29 (14.6%) | 0.002 | 26 (38.8%) | 14 (13.9%) | <0.001 |
| - MV duration (hours) | 169 (49, 369) | 95 (50, 161) | 0.21 | 150 (51, 310) | 83 (28, 118) | 0.047 |
| - Non-invasive ventilation (NIV) | 68 (48.9%) | 116 (57.7%) | 0.11 | 30 (44.1%) | 60 (57.7%) | 0.08 |
| - NIV duration (hours) | 15 (3, 15) | 14 (3, 43) | 0.57 | 12 (6, 41) | 18 (7, 32) | 0.84 |
| - Inotropes | 51 (36.2%) | 60 (29.7%) | 0.21 | 28 (41.8%) | 33 (32.0%) | 0.20 |
| - Renal replacement therapy | 9 (6.5%) | 13 (6.5%) | 0.98 | 4 (6.0%) | 4 (3.9%) | 0.53 |
| - ECMO | 1 (0.7%) | 0 (0) | 0.23 | 0 (0) | 0 (0) | - |
| - Tracheostomy | 6 (4.3%) | 3 (1.5%) | 0.11 | 1 (1.5%) | 1 (1.0%) | 0.76 |
| CFS – clinical frailty scale, SD – standard deviation, IQR. – interquartile range, BMI – body mass index, MET – medical emergency team, APACHE - Acute Physiology and Chronic Health Evaluation, ED – emergency department, ICU – intensive care unit, ROD – risk of death, ANZROD – Australia New Zealand risk of death | | | | | | |

**Supplementary Table 4:** Comparison of unadjusted secondary outcomes. Standard error bars are 95%CI.

| **Variable** | **Patients with  COVID-19** | **Patients without COVID-19** | **p-value** | **Patients with COVID-19 (Red)**  **Patients without COVID-19 (Black)** |
| --- | --- | --- | --- | --- |
| Hospital mortality overall, n (%) | 441 (14.7%) | 230 (14.9%) | 0.82 |  |
| Hospital mortality by CFS categories, n (%) | | | |  |
| - CFS-1-3 | 238/2,241 (10.6%) | 53/620 (8.5%) | 0.024 |  |
| - CFS-4 | 88/405 (21.7%) | 52/407 (12.8%) |  |  |
| - CFS-5 | 43/154 (27.9%) | 41/206 (19.9%) |  |  |
| - CFS-6 | 42/140 (30.0%) | 54/203 (26.6%) |  |  |
| - CFS-7-8 | 30/66 (45.5%) | 30/105 (28.6%) |  |  |
| ICU mortality overall, n (%) | 348 (11.4%) | 154 (10.0%) | 0.17 |  |
| ICU mortality by CFS categories, n (%) | | | |  |
| - CFS-1-3 | 197/2,289 (8.6%) | 36/620 (5.8%) | <0.001 |  |
| - CFS-4 | 70/410 (17.1%) | 36/406 (8.9%) |  |  |
| - CFS-5 | 33/156 (21.2%) | 29/205 (14.1%) |  |  |
| - CFS-6 | 27/143 (18.9%) | 31/202 (15.3%) |  |  |
| - CFS-7-8 | 21/67 (31.3%) | 21/67 (20.8%) |  |  |
| ICU length of stay overall, median (IQR) | 5.0 (2.1, 10.9) | 3.0 (1.6, 5.6) | <0.001 |  |
| ICU length of stay by CFS categories, n (%) | | | |  |
| - CFS-1-3 | 5.1 (2.3, 10.9) | 3.0 (1.6, 6.1) | <0.001 |  |
| - CFS-4 | 4.9 (2.0, 11.0) | 3.0 (1.5, 5.4) |  |  |
| - CFS-5 | 6.3 (1.9, 11.7) | 2.8 (1.5, 4.7) |  |  |
| - CFS-6 | 3.8 (1.6, 8.0) | 3.1 (1.7, 6.3) |  |  |
| - CFS-7-8 | 3.7 (1.8, 7.4) | 2.9 (1.3, 5.6) |  |  |
| Hospital length of stay overall, median (IQR) | 12.9 (7.4, 21.7) | 10.1 (5.4, 18.8) | <0.001 |  |
| Hospital length of stay by CFS categories, median (IQR) | | | |  |
| - CFS-1-3 | 12.7 (7.5, 21.2) | 9.0 (4.8, 17.2) | 0.91 |  |
| - CFS-4 | 13.6 (7.3, 24.0) | 10.9 (6.0, 21.2) |  |  |
| - CFS-5 | 16.5 (8.7, 24.4) | 10.4 (5.3, 18.9) |  |  |
| - CFS-6 | 12.2 (6.2, 21.9) | 11.0 (6.5, 20.0) |  |  |
| - CFS-7-8 | 8.3 (4.4, 18.7) | 10.0 (5.7, 19.2) |  |  |
| ICU Readmission overall, n (%) | 127 (4.1%) | 157 (10.2%) | <0.001 |  |
| ICU Readmission by CFS categories, n (%) | | | |  |
| - CFS-1-3 | 80/2,298 (3.5%) | 49/620 (7.9%) | <0.001 |  |
| - CFS-4 | 27/410 (6.6%) | 56/408 (13.7%) |  |  |
| - CFS-5 | 10/157 (6.4%) | 20/206 (9.7%) |  |  |
| - CFS-6 | 5/144 (3.5%) | 20/203 (9.9%) |  |  |
| - CFS-7-8 | 5/68 (7.4%) | 12/106 (11.3%) |  |  |
| Home discharge overall, n (%) | 1855 (60.3%) | 973 (63.1%) | 0.07 |  |
| Home discharge by CFS categories, n (%) | | | |  |
| - CFS-1-3 | 1,489/2,298 (64.8%) | 438/620 (70.6%) | <0.001 |  |
| - CFS-4 | 200/410 (48.8%) | 259/408 (63.5%) |  |  |
| - CFS-5 | 81/157 (51.6%) | 121/206 (58.7%) |  |  |
| - CFS-6 | 57/144 (39.6%) | 105/203 (51.7%) |  |  |
| - CFS-7-8 | 18/68 (26.5%) | 50/106 (47.2%) |  |  |
|  | | | | |
| New Nursing home discharge overall, n (%) | 19 (0.6%) | 34 (2.2%) | <0.001 |  |
| New Nursing home discharge by CFS categories, n (%) | | | |  |
| - CFS-1-3 | 5/2,298 (0.2%) | 7/620 (1.2%) | <0.001 |  |
| - CFS-4 | 4/410 (1.0%) | 9/410 (2.2%) |  |  |
| - CFS-5 | 3/157 (1.9%) | 4/206 (1.9%) |  |  |
| - CFS-6 | 3/144 (2.1%) | 7/203 (3.4%) |  |  |
| - CFS-7-8 | 4/68 (5.9%) | 7/106 (6.6%) |  |  |

**Supplementary Table 5:** Exposure and raw outcomes for male patients (2,679 patients [58.0%]; with COVID-19=1,887 patients; and without COVID-19=792 patients)**.**

| **Variable** | **COVID-19 status** | **CFS-1-3** | **CFS-4** | **CFS-5** | **CFS-6** | **CFS-7-8** | **p-value*** |  |
| --- | --- | --- | --- | --- | --- | --- | --- | --- |
| **EXPOSURE** | | | | | | | | |
| N | With COVID-19 | 1,435 | 248 | 88 | 68 | 48 | - |  |
|  | Without COVID-19 | 324 | 226 | 100 | 99 | 43 |  |  |
| Age, median (IQR) | With COVID-19 | 55.2 (43.5, 65.4) | 65.6 (56.3, 74.5) | 71.4 (62.1, 77.8) | 72.7 (64.4, 82.0) | 68.9 (52.4, 77.7) | <0.001 |  |
|  | Without COVID-19 | 60.8 (46.5, 71.9) | 68.1 (56.8, 75.9) | 68.5 (55.8, 79.4) | 75.7 (65.7, 82.6) | 67.9 (60.4, 77.2) |  |  |
| Mechanical ventilation | With COVID-19 | 668 (46.6%) | 109 (44.0%) | 40 (45.5%) | 22 (32.4%) | 22 (45.8%) | <0.001 |  |
|  | Without COVID-19 | 95 (29.3%) | 46 (19.9%) | 18 (18.0%) | 17 (17.2%) | 6 (14.0%) |  |  |
| ANZROD (%) (mean [SD]) | With COVID-19 | 8.0 [4.5] | 14.1 [9.4] | 18.0 [10.9] | 21.1 [19.7] | 24.6 [23.3] | <0.001 |  |
|  | Without COVID-19 | 11.1 [12.8] | 16.1 [9.3] | 20.0 [8.9] | 26.4 [22.6] | 29.1 [23.2] |  |  |
| **OUTCOMES** | | | | | | | | |
| Hospital mortality | With COVID-19 | 181 (12.6%) | 62 (25.0%) | 26 (29.5%) | 19 (27.9%) | 22 (45.8%) | <0.001 |  |
|  | Without COVID-19 | 32 (9.9%) | 30 (13.3%) | 22 (22.0%) | 31 (31.3%) | 14 (32.6%) |  |  |
| ICU mortality | With COVID-19 | 151 (10.6%) | 47 (19.0%) | 20 (23.0%) | 10 (14.7%) | 17 (36.2%) | <0.001 |  |
|  | Without COVID-19 | 22 (6.8%) | 22 (9.8%) | 18 (18.0%) | 18 (18.2%) | 12 (27.9%) |  |  |
| ICU length of stay (days), median (IQR) | With COVID-19 | 5.7 (2.6, 11.8) | 5.4 (2.0, 11.2) | 6.9 (1.7, 13.9) | 4.2 (1.7, 8.8) | 4.1 (2.0, 8.4) | <0.001 |  |
|  | Without COVID-19 | 3.5 (1.7, 7.1) | 3.2 (1.6, 5.6) | 2.8 (1.4, 5.1) | 3.6 (1.6, 7.1) | 3.1 (1.4, 6.0) |  |  |
| Hospital length of stay (days), median (IQR) | With COVID-19 | 13.0 (7.6, 22.3) | 13.6 (6.9, 23.9) | 16.9 (7.4, 24.9) | 11.8 (6.7, 22.0) | 9.3 (5.5, 22.4) | <0.001 |  |
|  | Without COVID-19 | 10.5 (5.5, 18.5) | 11.2 (6.4, 22.3) | 8.6 (4.3, 17.2) | 10.6 (6.8, 20.8) | 10.9 (4.2, 28.1) |  |  |
| ICU Readmission | With COVID-19 | 54 (3.8%) | 16 (6.5%) | 1 (1.1%) | 4 (5.9%) | 3 (6.3%) | <0.001 |  |
|  | Without COVID-19 | 27 (8.3%) | 29 (12.8%) | 1 (1.0%) | 14 (14.1%) | 7 (16.3%) |  |  |
| New Nursing home discharge | With COVID-19 | 4 (0.3%) | 3 (1.2%) | 1 (0.8%) | 2 (2.9%) | 3 (7.0%) | 0.022 |  |
|  | Without COVID-19 | 2 (0.6%) | 4 (1.8%) | 3 (2.8%) | 3 (3.0%) | 3 (7.0%) |  |  |
| Home discharge | With COVID-19 | 885 (61.7%) | 117 (47.2%) | 43 (48.9%) | 27 (39.7%) | 13 (27.1%) | 0.14 |  |
|  | Without COVID-19 | 221 (68.2%) | 139 (61.5%) | 58 (58.0%) | 46 (46.5%) | 16 (37.2%) |  |  |
| CFS – clinical frailty scale, ICU – intensive care unit, IQR – interquartile range, ANZROD – Australia and New Zealand risk of death score  *Comparison between patients with and without COVID-19 | | | | | | | | |

**Supplementary Table 6:** Exposure and raw outcomes for female patients (1941 patients [42.0%]; with COVID-19=1,190 patients; and without COVID-19=751 patients)**.**

| **Variable** | **COVID-19 status** | **CFS-1-3** | **CFS-4** | **CFS-5** | **CFS-6** | **CFS-7-8** | **p-value*** |  |
| --- | --- | --- | --- | --- | --- | --- | --- | --- |
| **EXPOSURE** | | | | | | | | |
| N | With COVID-19 | 863 | 162 | 69 | 76 | 20 | - |  |
|  | Without COVID-19 | 296 | 182 | 106 | 104 | 63 |  |  |
| Age, median (IQR) | With COVID-19 | 50.5 (37.1, 62.2) | 61.1 (49.2, 71.0) | 72.3 (63.0, 80.5) | 71.5 (62.6, 80.1) | 62.7 (57.5, 78.5) | <0.001 |  |
|  | Without COVID-19 | 58.8 (39.7, 70.8) | 65.1 (51.3, 78.0) | 70.3 (57.3, 79.2) | 72.6 (62.4, 80.7) | 74.5(65.7, 81.2) |  |  |
| Mechanical ventilation | With COVID-19 | 347 (40.2%) | 65 (40.1%) | 19 (27.5%) | 18 (23.7%) | 4 (20.0%) | <0.001 |  |
|  | Without COVID-19 | 67 (22.6%) | 37 (20.3%) | 18 (17.0%) | 12 (11.5%) | 8 (12.7%) |  |  |
| ANZROD (%) (mean [SD]) | With COVID-19 | 6.1 [7.1] | 11.4 [14.3] | 18.1 [18.7] | 20.2 [15.4] | 19.9 [21.1] | <0.001 |  |
|  | Without COVID-19 | 9.7 [13.8] | 15.9 [17.9] | 21.4 [20.7] | 19.0 [18.4] | 25.2 [21.5] |  |  |
| **OUTCOMES** | | | | | | | | |
| Hospital mortality | With COVID-19 | 57 (6.6%) | 26 (16.0%) | 17 (24.6%) | 23 (30.3%) | 8 (40.0%) | <0.001 |  |
|  | Without COVID-19 | 21 (7.1%) | 22 (12.1%) | 19 (17.9%) | 23 (22.1%) | 16 (25.4%) |  |  |
| ICU mortality | With COVID-19 | 46 (5.3%) | 23 (14.2%) | 13 (18.8%) | 17 (22.7%) | 4 (20.0%) | <0.001 |  |
|  | Without COVID-19 | 14 (4.7%) | 14 (7.7%) | 11 (10.5%) | 13 (12.6%) | 10 (15.9%) |  |  |
| ICU length of stay (days), median (IQR) | With COVID-19 | 4.5 (2.0, 9.9) | 4.4 (2.0, 11.0) | 6.1 (2.2, 10.1) | 3.8 (1.6, 7.3) | 2.6 (0.8, 5.7) | <0.001 |  |
|  | Without COVID-19 | 2.8 (1.6, 4.9) | 2.8 (1.6, 4.7) | 2.7 (1.6, 4.7) | 2.8 (1.7, 5.2) | 2.8 (1.3, 4.9) |  |  |
| Hospital length of stay (days), median (IQR) | With COVID-19 | 11.8 (7.2, 19.4) | 14.1 (8.1, 24.1) | 15.1 (9.2, 24.6) | 12.7 (6.0, 22.2) | 11.1 (6.4, 23.3) | <0.001 |  |
|  | Without COVID-19 | 8.1 (4.8, 16.7) | 10.2 (5.0, 20.1) | 11.6 (6.2, 20.9) | 11.5 (6.1, 18.0) | 11.9 (5.1, 16.8) |  |  |
| ICU Readmission | With COVID-19 | 26 (3.0%) | 11 (6.8%) | 7 (10.1%) | 1 (1.3%) | 2 (10.0%) | <0.001 |  |
|  | Without COVID-19 | 22 (7.4%) | 27 (14.8%) | 10 (9.4%) | 6 (5.8%) | 5 (7.9%) |  |  |
| New Nursing home discharge | With COVID-19 | 1 (0.1%) | 1 (0.6%) | 2 (2.9%) | 1 (1.3%) | 1 (5.0%) | <0.001 |  |
|  | Without COVID-19 | 5 (1.7%) | 5 (2.7%) | 3 (2.8%) | 4 (3.8%) | 4 (6.3%) |  |  |
| Home discharge | With COVID-19 | 604 (70.0%) | 93 (57.4%) | 38 (55.1%) | 30 (39.5%) | 5 (25.0%) | 0.67 |  |
|  | Without COVID-19 | 217 (73.3%) | 120 (65.9%) | 63 (59.4%) | 59 (56.7%) | 34 (54.0%) |  |  |
| CFS – clinical frailty scale, ICU – intensive care unit, IQR – interquartile range, ANZROD – Australia and New Zealand risk of death score  *Comparison between patients with and without COVID-19 | | | | | | | | |

**Supplementary Table 7:** Exposure and raw outcomes for patients ≥65 years (1,861 patients [40.3%]; with COVID-19=1,033 patients; and without COVID-19=828 patients).

| **Variable** | **COVID-19 status** | **CFS-1-3** | **CFS-4** | **CFS-5** | **CFS-6** | **CFS-7-8** | **p-value^*^** |
| --- | --- | --- | --- | --- | --- | --- | --- |
| **EXPOSURE** | | | | | | | |
| N | With COVID-19 | 580 | 199 | 112 | 105 | 37 | - |
|  | Without COVID-19 | 252 | 226 | 126 | 148 | 76 |  |
| Age, median (IQR) | With COVID-19 | 70.5 (67.4, 75.2) | 73.7 (69.1, 79.2) | 75.7 (70.8, 81.4) | 76.5 (70.7, 83.8) | 75.6 (70.3, 80.5) | 0.85 |
|  | Without COVID-19 | 73.7 (69.0, 78.9) | 75.4 (70.2, 80.4) | 77.1 (70.8, 82.4) | 77.8 (72.4, 83.9) | 76.1 (70.5, 81.6) |  |
| Male sex | With COVID-19 | 394 (67.9%) | 131 (65.8%) | 63 (56.3%) | 51 (48.6%) | 29 (76.8%) | <0.001 |
|  | Without COVID-19 | 138 (54.8%) | 131 (58.0%) | 58 (46.0%) | 76 (51.4%) | 28 (36.8%) |  |
| Mechanical ventilation | With COVID-19 | 281 (48.4%) | 71 (35.7%) | 38 (33.9%) | 22 (21.0%) | 13 (35.1%) | <0.001 |
|  | Without COVID-19 | 50 (19.8%) | 37 (16.4%) | 17 (13.5%) | 19 (12.8%) | 6 (7.9%) |  |
| ANZROD (%) (mean [SD]) | With COVID-19 | 9.4 [13.4] | 18.1 [15.7] | 21.3 [18.7] | 23.2 [17.5] | 33.6 [24.4] | 0.16 |
|  | Without COVID-19 | 14.9 [15.8] | 18.2 [15.8] | 24.6 [22.5] | 25.7 [21.6] | 29.6 [22.1] |  |
| **OUTCOMES** | | | | | | | |
| Hospital mortality | With COVID-19 | 153 (27.0%) | 68 (34.9%) | 36 (33.0%) | 35 (34.0%) | 20 (55.6%) | <0.001 |
|  | Without COVID-19 | 38 (15.1%) | 33 (14.6%) | 32 (25.4%) | 44 (29.7%) | 28 (36.8%) |  |
| ICU mortality | With COVID-19 | 122 (21.1%) | 51 (25.6%) | 29 (26.1%) | 21 (20.2%) | 13 (36.1%) | <0.001 |
|  | Without COVID-19 | 26 (10.3%) | 23 (10.2%) | 22 (17.6%) | 25 (17.0%) | 20 (26.3%) |  |
| ICU length of stay (days), median (IQR) | With COVID-19 | 7.1 (2.9, 14.3) | 4.6 (1.9, 10.0) | 5.7 (1.7, 11.7) | 3.0 (1.6, 6.9) | 3.5 (1.5, 6.7) | <0.001 |
|  | Without COVID-19 | 2.9 (1.5, 5.5) | 3.1 (1.6, 5.4) | 2.7 (1.5, 4.7) | 2.8 (1.5, 5.6) | 2.7 (1.3, 5.4) |  |
| Hospital length of stay (days), median (IQR) | With COVID-19 | 16.7 (9.7, 26.5) | 15.1 (8.1, 22.8) | 16.4 (8.5, 23.9) | 11.9 (5.4, 22.0) | 9.3 (5.4, 20.0) | 0.22 |
|  | Without COVID-19 | 11.5 (5.9, 18.9) | 12.2 (6.2, 22.1) | 10.8 (6.1, 17.1) | 10.7 (5.7, 17.8) | 11.3 (4.3, 19.2) |  |
| ICU Readmission | With COVID-19 | 831 (5.3%) | 12 (6.0%) | 8 (7.1%) | 3 (2.9%) | 3 (8.1%) | 0.002 |
|  | Without COVID-19 | 27 (10.7%) | 32 (14.2%) | 13 (10.3%) | 12 (8.1%) | 8 (10.5%) |  |
| New Nursing home discharge | With COVID-19 | 3 (0.5%) | 4 (2.0%) | 3 (2.7%) | 3 (2.9%) | 2 (5.4%) | 0.004 |
|  | Without COVID-19 | 5 (2.0%) | 7 (3.1%) | 3 (2.4%) | 6 (4.1%) | 6 (7.9%) |  |
| Home discharge | With COVID-19 | 265 (45.7%) | 85 (42.7%) | 54 (48.2%) | 35 (33.3%) | 6 (16.2%) | <0.001 |
|  | Without COVID-19 | 157 (62.3%) | 131 (58.0%) | 67 (53.2%) | 72 (48.6%) | 28 (36.8%) |  |
| CFS – clinical frailty scale, ICU – intensive care unit, IQR – interquartile range, ANZROD – Australia and New Zealand risk of death score  *Comparison between patients with and without COVID-19 | | | | | | | |

**Supplementary Table 8:** Exposure and raw outcomes in patients needing mechanical ventilation (1,642 patients [35.5%]; COVID-19=1,314 patients; non-COVID-19=328 patients).

| **Variable** | **COVID-19 status** | **CFS-1-3** | **CFS-4** | **CFS-5** | **CFS-6** | **CFS-7-8** | **p-value^*^** |
| --- | --- | --- | --- | --- | --- | --- | --- |
| **EXPOSURE** | | | | | | | |
| N | With COVID-19 | 1015 | 174 | 59 | 40 | 26 | - |
|  | Without COVID-19 | 162 | 87 | 36 | 29 | 14 |  |
| Age, median (IQR) | With COVID-19 | 56.1 (45.9, 65.2) | 60.8 (49.5, 69.7) | 69.3 (57.9, 74.9) | 66.4 (56.4, 74.4) | 63.7 (53.4, 73.8) | <0.001 |
|  | Without COVID-19 | 56.6 (44.1, 67.9) | 62.4 (45.3, 73.7) | 61.4 (44.5, 73.8) | 68.8 (59.6, 76.2) | 60.5 (47.1, 70.3) |  |
| Male sex | With COVID-19 | 668 (65.8%) | 109 (62.6%) | 40 (67.8%) | 22 (55.0%) | 22 (84.6%) | 0.037 |
|  | Without COVID-19 | 95 (58.6%) | 50 (57.5%) | 18 (50.0%) | 17 (58.6%) | 6 (42.9%) |  |
| ANZROD (%) (mean [SD]) | With COVID-19 | 10.0 [11.0] | 15.7 [15.7] | 20.5 [19.1] | 19.6 [19.4] | 23.7 [22.6] | <0.001 |
|  | Without COVID-19 | 14.4 [14.1] | 23.0 [20.3] | 26.1 [20.1] | 31.9 [25.2] | 28.3 [27.4] |  |
| **OUTCOMES** | | | | | | | |
| Hospital mortality | With COVID-19 | 184 (18.6%) | 52 (30.1%) | 19 (32.2%) | 12 (31.6%) | 16 (61.5%) | <0.001 |
|  | Without COVID-19 | 25 (15.4%) | 23 (26.4%) | 12 (33.3%) | 13 (44.8%) | 5 (35.7%) |  |
| ICU mortality | With COVID-19 | 171 (16.9%) | 49 (28.2%) | 18 (30.5%) | 9 (23.1%) | 14 (53.8%) | <0.001 |
|  | Without COVID-19 | 22 (13.6%) | 15 (17.2%) | 10 (27.8%) | 10 (34.5%) | 4 (28.6%) |  |
| ICU length of stay (days), median (IQR) | With COVID-19 | 10.9 (5.8, 18.8) | 11.2 (5.0, 20.8) | 12.9 (7.0, 19.9) | 12.0 (3.8, 20.6) | 8.4 (5.0, 14.1) | 0.23 |
|  | Without COVID-19 | 7.7 (3.7, 14.2) | 8.6 (4.9, 15.3) | 8.4 (4.5, 13.3) | 7.8 (4.5, 11.8) | 6.6 (2.0, 11.2) |  |
| Hospital length of stay (days), median (IQR) | With COVID-19 | 19.8 (11.6, 32.8) | 16.2 (8.9, 28.7) | 10.6 (5.7, 15.9) | 19.4 (1.1, 41.5) | 9.3 (5.7, 19.9) | 0.08 |
|  | Without COVID-19 | 17.1 (10.1-29.4) | 17.2 (8.1, 30.0) | 20.4 (14.3, 28.8) | 17.3 (8.6-30.6) | 15.2 (6.6, 23.7) |  |
| ICU Readmission | With COVID-19 | 28 (2.8%) | 6 (3.4%) | 2 (3.4%) | 1 (2.5%) | 2 (7.7%) | <0.001 |
|  | Without COVID-19 | 11 (6.8%) | 15 (17.2%) | 3 (8.3%) | 3 (10.3%) | 1 (7.1%) |  |
| New Nursing home discharge | With COVID-19 | 2 (0.2%) | 1 (0.6%) | 0 (0) | 0 (0) | 0 (0) | 0.27 |
|  | Without COVID-19 | 3 (1.9%) | 1 (1.1%) | 0 (0) | 0 (0) | 0 (0) |  |
| Home discharge | With COVID-19 | 407 (40.1%) | 53 (30.5%) | 25 (42.4%) | 12 (30.0%) | 4 (15.4%) | 0.003 |
|  | Without COVID-19 | 83 (51.2%) | 39 (44.8%) | 14 (38.9%) | 10 (34.5%) | 6 (42.9%) |  |
| CFS – clinical frailty scale, ICU – intensive care unit, IQR – interquartile range, ANZROD – Australia and New Zealand risk of death score  *Comparison between patients with and without COVID-19  **No statistics could be computed. | | | | | | | |

**Supplementary Table 9:** Exposure and raw outcomes for patients who were cared for in the year 2020 (1,163 patients [25.2%]; with COVID-19=444 patients; and without COVID-19=719 patients).

| **Variable** | **COVID-19 status** | **CFS-1-3** | **CFS-4** | **CFS-5** | **CFS-6** | **CFS-7-8** | **p-value^*^** |
| --- | --- | --- | --- | --- | --- | --- | --- |
| **EXPOSURE** | | | | | | | |
| N | With COVID-19 | 317 | 85 | 35 | 35 | 19 | - |
|  | Without COVID-19 | 270 | 177 | 98 | 90 | 37 |  |
| Age, median (IQR) | With COVID-19 | 59.3 (48.0, 70.7) | 72.5 (60.3, 79.2) | 73.2 (60.6, 80.8) | 72.5 (67.1, 85.0) | 75.6 (70.3, 80.5) | <0.001 |
|  | Without COVID-19 | 58.8 (41.3, 71.1) | 66.0 (54.2, 76.0) | 66.8 (55.4, 79.2) | 72.5 (61.6, 80.9) | 76.1 (70.5, 81.6) |  |
| Male sex | With COVID-19 | 207 (65.3%) | 52 (61.2%) | 20 (57.1%) | 16 (45.7%) | 10 (52.6%) | 0.002 |
|  | Without COVID-19 | 133 (49.3%) | 94 (53.1%) | 50 (51.0%) | 42 (46.7%) | 15 (40.5%) |  |
| Mechanical ventilation | With COVID-19 | 139 (43.8%) | 33 (38.8%) | 11 (31.4%) | 10 (28.6%) | 7 (36.8%) | <0.001 |
|  | Without COVID-19 | 69 (25.6%) | 41 (23.2%) | 14 (14.3%) | 18 (20.0%) | 6 (16.2%) |  |
| ANZROD (%) (mean [SD]) | With COVID-19 | 8.0 [10.4] | 14.3 [13.8] | 14.9 [19.8] | 21.8 [20.3] | 33.6 [24.4] | <0.001 |
|  | Without COVID-19 | 8.9 [12.7] | 15.2 [16.4] | 19.5 [21.1] | 24.4 [22.8] | 29.6 [22.1] |  |
| **OUTCOMES** | | | | | | | |
| Hospital mortality | With COVID-19 | 27 (8.5%) | 16 (18.8%) | 9 (25.7%) | 10 (28.6%) | 5 (26.3%) | <0.001 |
|  | Without COVID-19 | 19 (7.0%) | 20 (11.3%) | 18 (18.4%) | 23 (25.6%) | 10 (27.0%) |  |
| ICU mortality | With COVID-19 | 21 (6.6%) | 14 (16.5%) | 4 (11.4%) | 6 (17.1%) | 3 (15.8%) | 0.011 |
|  | Without COVID-19 | 17 (6.3%) | 12 (6.8%) | 11 (11.2%) | 13 (14.4%) | 6 (16.2%) |  |
| ICU length of stay (days), median (IQR) | With COVID-19 | 4.3 (1.9, 11.3) | 3.2 (1.6, 8.7) | 3.9 (1.4, 10.7) | 1.7 (0.9, 4.4) | 3.5 (1.5, 6.7) | 0.005 |
|  | Without COVID-19 | 2.9 (1.7, 5.6) | 3.0 (1.5, 5.3) | 2.9 (1.5, 4.7) | 2.9 (1.4, 6.5) | 2.7 (1.3, 5.4) |  |
| Hospital length of stay (days), median (IQR) | With COVID-19 | 11.9 (5.9, 21.2) | 11.9 (6.0, 23.1) | 12.5 (6.0, 20.3) | 10.5 (4.4, 23.6) | 9.3 (5.4, 20.0) | 0.44 |
|  | Without COVID-19 | 8.1 (5.0, 15.2) | 10.4 (5.2, 20.0) | 10.0 (4.6, 17.4) | 10.0 (5.4, 18.1) | 11.3 (4.3, 19.2) |  |
| ICU Readmission | With COVID-19 | 11 (3.5%) | 7 (8.2%) | 0 (0) | 1 (2.9%) | 0 (0) | 0.004 |
|  | Without COVID-19 | 18 (6.7%) | 23 (13.0%) | 9 (9.2%) | 7 (7.8%) | 5 (13.5%) |  |
| New Nursing home discharge | With COVID-19 | 3 (0.9%) | 2 (2.4%) | 2 (5.7%) | 2 (5.7%) | 2 (10.5%) | 0.001 |
|  | Without COVID-19 | 3 (1.1%) | 2 (1.1%) | 1 (1.0%) | 5 (5.6%) | 3 (8.1%) |  |
| Home discharge | With COVID-19 | 213 (67.2%) | 48 (56.5%) | 20 (57.1%) | 11 (31.4%) | 5 (26.3%) | <0.001 |
|  | Without COVID-19 | 198 (73.3%) | 118 (66.7%) | 63 (64.3%) | 45 (50.0%) | 18 (48.6%) |  |
| CFS – clinical frailty scale, ICU – intensive care unit, IQR – interquartile range, ANZROD – Australia and New Zealand risk of death score  ^*^Comparison between patients with and without COVID-19 | | | | | | | |

**Supplementary Table 10:** Exposure and raw outcomes for patients who were cared for in the year 2021 (3,457 patients [74.8%]; with COVID-19=2,942 patients; and without COVID-19=972 patients)**.**

| **Variable** | **COVID-19 status** | **CFS-1-3** | **CFS-4** | **CFS-5** | **CFS-6** | **CFS-7-8** | **p-value*** |  |
| --- | --- | --- | --- | --- | --- | --- | --- | --- |
| **EXPOSURE** | | | | | | | | |
| N | With COVID-19 | 1,981 | 231 | 122 | 109 | 49 | - |  |
|  | Without COVID-19 | 350 | 325 | 108 | 113 | 76 |  |  |
| Age, median (IQR) | With COVID-19 | 52.4 (40.0, 63.4) | 62.2 (50.6, 71.4) | 71.2 (63.0, 77.9) | 71.0 (63.6, 80.1) | 65.4 (52.3, 74.2) | <0.001 |  |
|  | Without COVID-19 | 60.2 (45.8,71.7) | 67.0 (52.5, 76.9) | 70.5 (58.7, 79.4) | 75.0 (66.3, 82.7) | 73.8 (62.7, 80.2) |  |  |
| Male sex | With COVID-19 | 1228 (62.0%) | 196 (60.3%) | 68 (55.7%) | 52 (47.7%) | 38 (77.6%) | <0.001 |  |
|  | Without COVID-19 | 191 (54.6%) | 132 (57.1%) | 50 (46.3%) | 57 (50.4%) | 28 (40.6%) |  |  |
| Mechanical ventilation | With COVID-19 | 876 (44.2%) | 141 (43.7%) | 48 (39.3%) | 30 (27.5%) | 19 (38.8%) | <0.001 |  |
|  | Without COVID-19 | 93 (26.6%) | 46 (19.9%) | 22 (20.4%) | 11 (9.7%) | 8 (11.6%) |  |  |
| ANZROD (%) (mean [SD]) | With COVID-19 | 7.2 [9.0] | 12.7 [14.3] | 19.0 [17.6] | 20.3 [16.6] | 24.0 [25.0] | <0.001 |  |
|  | Without COVID-19 | 11.5 [13.6] | 16.7 [18.5] | 21.5 [20.8] | 21.2 [19.3] | 27.0 [21.4] |  |  |
| **OUTCOMES** | | | | | | | | |
| Hospital mortality | With COVID-19 | 211 (11.0%) | 72 (22.5%) | 34 (28.6%) | 32 (30.5%) | 25 (53.2%) | <0.001 |  |
|  | Without COVID-19 | 34 (9.7%) | 32 (13.9%) | 23 (21.3%) | 31 (27.4%) | 20 (29.4%) |  |  |
| ICU mortality | With COVID-19 | 176 (8.9%) | 56 (17.2%) | 29 (24.0%) | 21 (19.4%) | 18 (37.5%) | <0.001 |  |
|  | Without COVID-19 | 19 (5.4%) | 23 (10.4%) | 18 (16.8%) | 18 (16.1%) | 16 (23.2%) |  |  |
| ICU length of stay (days), median (IQR) | With COVID-19 | 5.2 (2.4, 10.9) | 5.4 (2.1, 11.8) | 7.0 (2.3, 12.8) | 4.3 (2.1, 8.1) | 3.6 (1.8, 9.2) | <0.001 |  |
|  | Without COVID-19 | 3.1 (1.6, 6.4) | 2.9 (1.6, 5.6) | 2.6 (1.5, 4.7) | 3.5 (1.8, 6.3) | 2.7 (1.3, 4.9) |  |  |
| Hospital length of stay (days), median (IQR) | With COVID-19 | 12.7 (7.8, 21.1) | 14.0 (7.5, 24.1) | 17.0 (9.8, 26.9) | 12.7 (7.2, 21.3) | 12.9 (6.1, 23.9) | 0.61 |  |
|  | Without COVID-19 | 9.5 (4.7, 18.9) | 11.2 (6.4, 22.1) | 10.9 (5.4, 20.3) | 12.7 (7.2, 20.1) | 12.7 (4.6, 18.5) |  |  |
| ICU Readmission | With COVID-19 | 69 (3.5%) | 20 (6.2%) | 10 (8.2%) | 4 (3.7%) | 5 (10.2%) | <0.001 |  |
|  | Without COVID-19 | 31 (8.9%) | 33 (14.3%) | 11 (10.2%) | 13 (11.5%) | 7 (10.1%) |  |  |
| New Nursing home discharge | With COVID-19 | 2 (0.1%) | 2 (0.6%) | 1 (0.8%) | 1 (0.9%) | 2 (4.1%) | <0.001 |  |
|  | Without COVID-19 | 4 (1.1%) | 7 (3.0%) | 3 (2.8%) | 2 (1.8%) | 4 (5.8%) |  |  |
| Home discharge | With COVID-19 | 1,276 (64.4%) | 162 (49.8%) | 61 (50.0%) | 46 (42.2%) | 13 (26.5%) | <0.001 |  |
|  | Without COVID-19 | 240 (64.4%) | 141 (61.0%) | 58 (53.7%) | 60 (53.1%) | 32 (46.4%) |  |  |
| CFS – clinical frailty scale, ICU – intensive care unit, IQR – interquartile range, ANZROD – Australia and New Zealand risk of death score  *Comparison between patients with and without COVID-19 | | | | | | | | |

**Supplementary Table 11:** Predictors for hospital mortality with the Clinical Frailty Scale (CFS) categories, adjusted for ANZROD and male sex, except for biological sex that was adjusted only for ANZROD.

| **Predictor** | **Patients with COVID-19** | **Patients without COVID-19** |
| --- | --- | --- |
|  | **OR (95%CI)** | **OR (95%CI)** |
| **All patients** | | |
| - CFS-1-3 | Reference | Reference |
| - CFS-4 | 1.59 (1.17-2.15) | 1.11 (0.71-1.71) |
| - CFS-5 | 1.69 (1.09-2.61) | 1.56 (0.95-2.56) |
| - CFS-6 | 1.57 (0.99-2.48) | 2.24 (1.40-3.56) |
| - CFS-7-8 | 3.08 (1.70-5.59) | 2.04 (1.14-3.62) |
| Male sex | 1.53 (1.19-1.95) | 1.28 (0.93-1.76) |
| ANZROD | 1.07 (1.06-1.08) | 1.05 (1.04-1.06) |
| **Age ≥65 years** | | |
| - CFS-1-3 | Reference | Reference |
| - CFS-4 | 1.22 (0.84-1.76) | 0.78 (0.46-1.34) |
| - CFS-5 | 0.99 (0.61-1.59) | 1.27 (0.71-2.27) |
| - CFS-6 | 0.96 (0.59-1.57) | 1.58 (0.92-2.69) |
| - CFS-7-8 | 1.62 (0.76-3.49) | 1.98 (1.05-3.73) |
| Male sex | 1.34 (1.03-1.83) | 1.04 (0.72-1.51) |
| ANZROD | 1.04 (1.03-1.06) | 1.04 (1.03-1.05) |
| **Male sex*** | | |
| - CFS-1-3 | Reference | Reference |
| - CFS-4 | 1.02 (0.52-2.00) | 1.00 (0.56-1.78) |
| - CFS-5 | 1.56 (0.89-2.73) | 1.59 (0.82-3.09) |
| - CFS-6 | 1.51 (1.05-2.18) | 2.02 (1.07-3.80) |
| - CFS-7-8 | 2.60 (1.26-5.34) | 1.87 (0.81-4.33) |
| ANZROD | 1.06 (1.05-1.07) | 1.04 (1.03-1.05) |
| **Female sex*** | | |
| - CFS-1-3 | Reference | Reference |
| - CFS-4 | 1.84 (1.07-3.16) | 1.27 (0.64-2.52) |
| - CFS-5 | 2.12 (1.05-4.29) | 1.56 (0.75-3.27) |
| - CFS-6 | 2.69 (1.41-5.12) | 2.54 (1.27-5.10) |
| - CFS-7-8 | 4.63 (1.62-13.29) | 2.24 (1.00-5.02) |
| ANZROD | 1.06 (1.05-1.07) | 1.04 (1.03-1.05) |
| **Mechanical ventilation** | | |
| - CFS-1-3 | Reference | Reference |
| - CFS-4 | 1.37 (0.92-2.04) | 1.42 (0.72-2.81) |
| - CFS-5 | 1.14 (0.60-2.18) | 1.86 (0.78-4.46) |
| - CFS-6 | 1.17 (0.53-2.60) | 2.54 (0.99-6.49) |
| - CFS-7-8 | 4.18 (1.71-10.19) | 1.94 (0.52-7.23) |
| Male sex | 1.34 (0.98-1.84) | 1.25 (0.71-2.19) |
| ANZROD | 1.06 (1.05-1.07) | 1.04 (1.03-1.05) |
| **The year 2020** | | |
| - CFS-1-3 | Reference | Reference |
| - CFS-4 | 1.65 (0.77-3.52) | 1.16 (0.57-2.38) |
| - CFS-5 | 3.17 (1.17-8.54) | 1.62 (0.74-3.54) |
| - CFS-6 | 2.32 (0.83-6.50) | 2.07 (0.96-4.45) |
| - CFS-7-8 | 1.90 (0.53-6.81) | 2.11 (0.78-5.75) |
| Male sex | 2.89 (1.43-5.90) | 1.39 (0.83-2.32) |
| ANZROD | 1.07 (1.05-1.09) | 1.05 (1.04-1.06) |
| **The year 2021** | | |
| - CFS-1-3 | Reference | Reference |
| - CFS-4 | 1.63 (1.17-2.28) | 1.08 (0.62-1.88) |
| - CFS-5 | 1.49 (0.91-2.44) | 1.54 (0.81-2.93) |
| - CFS-6 | 1.49 (0.89-2.50) | 2.38 (1.32-4.29) |
| - CFS-7-8 | 4.32 (2.14-8.69) | 1.97 (0.97-3.98) |
| Male sex | 1.37 (1.06-1.79) | 1.21 (0.80-1.81) |
| ANZROD | 1.07 (1.06-1.08) | 1.05 (1.04-1.06) |
| CFS = clinical frailty scale, ANZROD = Australia and New Zealand risk of death  * Adjusted for ANZROD | | |
